# Histones and their chaperones: Adaptive remodelers of an ever-changing chromatinic landscape

**DOI:** 10.3389/fgene.2022.1057846

**Published:** 2022-11-16

**Authors:** Karla Torres-Arciga, Manuel Flores-León, Samuel Ruiz-Pérez, Magalli Trujillo-Pineda, Rodrigo González-Barrios, Luis A. Herrera

**Affiliations:** ^1^ Doctorado en Ciencias Biológicas, Universidad Nacional Autónoma de México (UNAM), Mexico City, Mexico; ^2^ Unidad de Investigación Biomédica en Cáncer, Instituto Nacional de Cancerología (INCan)-Instituto de Investigaciones Biomédicas (IIBO), Universidad Nacional Autónoma de México (UNAM), Mexico City, Mexico; ^3^ Departamento de Medicina Genómica y Toxicología Ambiental, Instituto de Investigaciones Biomédicas (IIBO), Universidad Nacional Autónoma de México (UNAM), Mexico City, Mexico; ^4^ Instituto Nacional de Medicina Genómica (INMEGEN), Mexico City, Mexico

**Keywords:** epigenetics, histone variants, transposons, aging, syndromes, chromatin remodeling, nucleosome

## Abstract

Chromatin maintenance and remodeling are processes that take place alongside DNA repair, replication, or transcription to ensure the survival and adaptability of a cell. The environment and the needs of the cell dictate how chromatin is remodeled; particularly where and which histones are deposited, thus changing the canonical histone array to regulate chromatin structure and gene expression. Chromatin is highly dynamic, and histone variants and their chaperones play a crucial role in maintaining the epigenetic regulation at different genomic regions. Despite the large number of histone variants reported to date, studies on their roles in physiological processes and pathologies are emerging but continue to be scarce. Here, we present recent advances in the research on histone variants and their chaperones, with a focus on their importance in molecular mechanisms such as replication, transcription, and DNA damage repair. Additionally, we discuss the emerging role they have in transposable element regulation, aging, and chromatin remodeling syndromes. Finally, we describe currently used methods and their limitations in the study of these proteins and highlight the importance of improving the experimental approaches to further understand this epigenetic machinery.

## Introduction

Chromatin protects and regulates the DNA; it is also tightly involved in nuclear processes that ensure the proper functionality of the cell. The basic unit of the chromatin is the nucleosome, composed of an octamer of histones (H2A, H2B, H3, and H4) that surround 146 base pairs (bp) of DNA (Luger et al., 1997). The role of histones is not restricted to merely core components of the nucleosome, they are key elements in chromatin organization inside the nucleus, providing dynamism to the so-called chromatin states: heterochromatin and euchromatin (Felsenfeld and Groudine, 2003). Nevertheless, it should be noted that changes in chromatin states are also dictated or influenced by other epigenetic components such as post-translational modifications (PTMs) that are present in histones and their variants, DNA modifications (methylation, 5-hydroxymethylation, etc.), insulators, chromatin remodeling complexes, among others. For each of the canonical histones, there are histone variants that not only make the epigenetic code more extensive and complex but also help to delimit chromatin states and processes.

Although during the last 10 years the study of histone variants, particularly of H3 and H2A, and their chaperones has increased considerably, the majority of these studies have focused on a few cellular processes and pathologies, resulting in an imbalanced knowledge and scattered information. Given this, in the present work, we aim to focus on the involvement of histone variants, particularly H3 and H2A, and their chaperones in distinct processes, highlighting their adaptiveness as an important component of replication, transcription, DNA repair and regulation of transposable elements (Table 1). For more detailed information regarding the histone variants including those of H1 and H2B, please refer to Fyodorov et al. (2018), Buschbeck and Hake (2017), and Martire and Banaszynski (2020). Furthermore, we briefly discuss their role through aging and in some syndromes. Finally, we present some challenges and useful techniques used in the study of these molecules.

**TABLE 1:** Principal functions of H2A and H3 variants.

Canonical histone	Histone variant	Histone chaperone/Chromatin remodeler	Function	References
H2A	H2A.Z	ANP32E	Related to activation and repression of transcription	Gursoy-Yuzugullu et al. (2015); Xu et al. (2012); Alatwi and Downs (2015); Begum et al. (2021)
		INO80		
		P400		
		SRCAP		
	H2A.J	Unknown	Implicated in the induction of the senescence phenotype	Contrepois et al. (2017)
	H2A.X	FACT	Signaling of DNA Damage Response	Piquet et al. (2018)
	MacroH2A	LSH	Chromatin compaction and transcriptional repression	Xu et al. (2021)
		FACT		
	H2A.B	Unknown	Mark replication origins and promotes splicing	Sansoni et al. (2014); Soboleva et al. (2017)
H3	H3.3	HIRA (HUCA complex)	Related to both euchromatin and heterochromatin maintenance	Elsaesser and Allis (2010)
		DAXX-ATRX		
	CENPA	HJURP	Maintenance of centromeres and chromosome stability	Yilmaz et al. (2021)

## Histone variants and histones chaperones: General concepts

In order to maintain the structure of the chromatin, histones play a critical role. Two broad types of histones have been described. The first type comprises what are commonly known as canonical histones (also introduced as replication-coupled histones), and the second class encompasses histone variants (also named as replication-independent histones). Replication-coupled histones’ genes are encoded in clusters (multiple-copies of the same canonical histone gene), they are intronless, their messenger RNAs (mRNAs) have a special 3′-loop end and are especially transcribed and deposited during the DNA synthesis (S) phase (Marzluff et al., 2008). Meanwhile, replication-independent histones are encoded in distinct isolated genes, may contain introns, their mRNA incorporates a polyA tail, and their expression and deposition occur throughout the cell cycle (Buschbeck and Hake, 2017). Histone variants can vary from just four amino acids (in the case of H3.3 in comparison with replication-coupled H3.2) to hundreds of different residues (for example, the macrodomain present in MacroH2A). These differences in amino acid composition confer distinct properties to the nucleosome; for example, by being the target of different post-translational modifications (PTMs) or interacting with distinct proteins or even with the DNA (Venkatesh and Workman, 2015). This diversity allows for a bigger and broader epigenetic regulation through increasing the possible PTMs at their N-terminal domain, as well as conferring distinct physicochemical properties to the nucleosome, hence allowing the formation of different protein complexes associated with the chromatin (Martire and Banaszynski, 2020).

Through the cell cycle, the different types of histones are incorporated into the chromatin as a response to internal and external stimuli. The proteins that deposit histones on DNA are called histone chaperones. Traditionally, a protein was considered a histone chaperone if it had the ability to bind histones and deposit them *in vitro* without the need for ATP (De Koning et al., 2007). However, nowadays, a protein is considered a histone chaperone if it has the ability to specifically bind and protect positively charged histones from interacting erroneously with other histones and DNA without having to hydrolyze ATP (Grover et al., 2018). Histone chaperones can be further classified into two broad groups: “dedicated” and “casual.” The former only recognize and interact with one specific histone, whereas the latter, due to their low selectivity, can interact with distinct histones (Jeffery et al., 2019). For more detailed histone chaperone reviews, please refer to the ones published by Gurard-Levin et al. (2014) and Hammond et al. (2017).

## The triad of nucleosomes disruption

**FIGURE 1:**
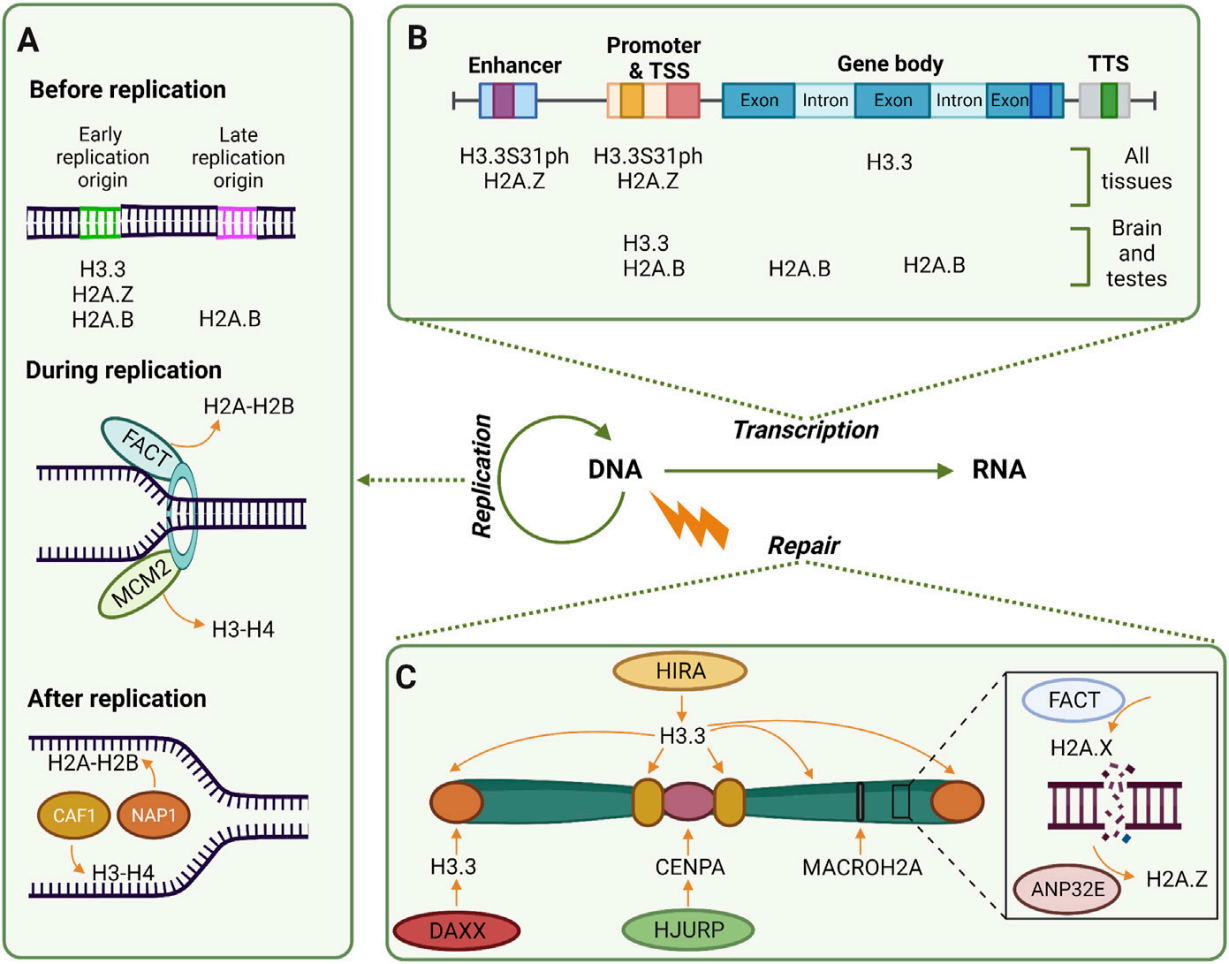
Histone variants are largely involved in replication, transcription, and replication**. (A)** Before replication, early replication origins are enriched in H3.3 and H2A.Z in the majority of the cells, while H2A.B is in both early and late replication origins in the brain and testes. During replication, chaperones FACT and MCM2 remove histones to have naked DNA ready. Right after replication, chaperones CAF1 and NAP1 deposit replication dependent H3-H4 and H2A-H2B respectively. **(B)** Transcription active genes are enriched with different histone variants. While H3.3 and H2A.Z are enriched at enhancers and promoters, H3.3 is also present at gene bodies. H2A.B is present at promoters and intron-exon boundaries of active genes of the brain and testes. **(C)** Along the chromosome, distinct variants are deposited by distinct chaperones depending on the regions and the DDR pathway. For example, when UVC damages DNA, H2A.Z is evicted from these sites by ANP32E and is substituted by H2A.X deposited by FACT; in centromeres HJURP deposits CENPA when DSB occurs at G1; deposition of macroH2A occurs when facultative heterochromatin is damaged, meanwhile, H3.3 is deposited in damaged telomeres by DAXX and ATRX (not depicted). Along the chromosome, including heterochromatic regions, HIRA deposits H3.3 in UVC-damaged chromatin.

Although proper distribution and positioning of the nucleosomes are extremely important for cell homeostasis, essential processes in the nucleus such as replication, transcription, and repair convey extensive disruption and remodeling of the chromatin (Figure 1). As such, it is worth discussing what is known so far about the histones variants and chaperones involved in these processes.

### Histone variants mark DNA replication origins

DNA replication represents a highly disruptive process for chromatin; therefore, tight regulation of this process and its components, including histones, is necessary. Although it is known that replication-coupled histones are deposited during the S phase to ensure that a sufficient or equal amount of histones is present in the chromatin after DNA is synthesized (Mei et al., 2017), the involvement of replication-independent histones during DNA replication has been studied to a lesser extent. Two main variants have been found to be enriched at replication origins, marking, and insulating these regions (Figure 1A). The first one is H3.3, a well-studied variant involved in DNA replication, which particularly marks the chromatin that replicates early employing two different deposition machineries. When recycling parental H3.3 during S phase, it is the histone chaperone ASF1 (anti-silencing function 1) that deposits the variant at the replication sites, forming domains that gradually decrease in quantity as the replication occurs and H3.1 takes over (Clément et al., 2018). In contrast, during *de novo* deposition of H3.3 on replicating chromatin, Gatto et al. (2022) demonstrated that the histone chaperone HIRA (histone cell cycle regulator) deposits H3.3 during S phase at previously defined early replication zones. These regions are characterized by an enrichment of H3.3 surrounded by H3.1 at the borders, constituting a boundary that is re-established despite replication. These reports provide evidence of distinct mechanisms that help preserve the epigenome even when the integrity of the chromatin becomes compromised due to disruptive processes such as replication.

The second replication-uncoupled histone described in DNA replication is H2A.Z. This histone is also incorporated at early replication origins, and its deposition at these sites prompts faster origin firing in comparison with nucleosomes containing canonical H2A. H2A.Z acts by recruiting SUV420H1 to establish H4K20me2 at these nucleosomes, which in turn recruits ORC1 to bind to these replication origins (Long et al., 2020). Known to a lesser extent is H2A.B, previously known as H2A.Bdd, which can also be deposited at origins, both early and late replication sites. Interestingly, it was observed that the presence of H2A.B is limited to the first 60 min after DNA is synthesized, however, its function has yet to be elucidated, specifically considering that only a few tissues, such as testis and brain, overexpress this variant (Sansoni et al., 2014). Nevertheless, the mechanism of incorporation of these replication-uncoupled H2A variants and the signals triggering their deposition at replication sites is still unknown. Further efforts are necessary to determine the overlap of H3.3 and H2A.Z nucleosomes in early replication origins, in order to understand their role as epigenetic regulators at these sites.

In certain genomic loci, replication proves to be a challenge due to the inherent characteristics of these regions such as the formation of secondary DNA structures, tightly bound protein complexes or highly compacted chromatin. Notably, Xu et al. (2021) observed that when Helicase, Lymphoid Specific (LSH), a chromatin remodeler involved in the incorporation of MacroH2A, is missing, cells become more prone to manifest replication stress and increased genomic instability. Interestingly, the loss of MacroH2A results in a similar phenotype suggesting that both LSH and MacroH2A play a significant role in maintaining the chromatin in telomeres and satellite regions, preventing replication fork stalling.

Once replication has started, nucleosomes are evicted at the replication fork by distinct proteins such as the histone chaperone FACT (facilitates chromatin transcription) complex and the MCM2 (minichromosome maintenance complex component 2). Different studies have shown that the SPT16 (suppressor of Ty 16) subunit of the FACT complex displaces H2A-H2B dimers, while the other subunit, the structure-specific recognition protein 1 (SSRP1), stabilizes the H3-H4 tetramer (Chen et al., 2018; Wang et al., 2018; Liu et al., 2020). The ability of FACT to unfold nucleosomes, not only in replication but also in transcription, is further enhanced by its interaction with Nhp6 (non-histone chromosomal protein 6A). It does so by exposing the histone-binding sites in FACT before its interaction with the nucleosome, allowing it to assume a more open conformation (Sivkina et al., 2022). Meanwhile, MCM2, a subunit of the replicative helicase, interacts with the H3-H4 tetramer, preventing the histones from associating with the DNA. Interestingly, Huang et al. (2015) observed that human MCM2 is also capable of interacting with H3.3 as well as with CENPA (histone H3-like centromeric protein A); in fact, MCM2 can associate with both the replication-coupled H3.1 and H3.2, as well as the replication-uncoupled H3.3 (Latreille et al., 2014; Huang et al., 2015). Such findings suggest that MCM2 and ASF1 could cochaperone, and together participate in the redeposition and recycling of H3-H4 dimers. This supports the existence of a general mechanism to rapidly remove and reposition histones after the replication fork.

### Histone variants are differently positioned throughout active genes to enable transcription

The cell differentiates between transcription states not only by the histone PTMs, but also by differentially depositing histone variants at particular genomic regions (Figure 1B). This is the case of H3.3, one of the best studied cases of histone variants in transcription. This replication uncoupled histone is enriched at enhancers, promoters, and gene bodies of active genes (Schwartz and Ahmad, 2005; Chen et al., 2013). The chaperone that deposits the variant at these regions is HIRA, and it was recently shown that depending on whether the H3.3 to be deposited is newly synthesized or recycled, the composition of the HIRA complex differs. For new histones the trimerization of HIRA and its interaction with UBN1 (ubinuclein 1) are required, while for recycled histones the complex does not need HIRA trimerization or UBN1 interaction but is dependent on interaction of HIRA with ASF1 (Torné et al., 2020). This fine-tuning model of balancing old vs. new histones through histone chaperone complexes formation is worth exploring in other chaperone complexes as well as in other nuclear processes.

Histone variants H3.1 and H3.2 show a high similarity to H3.3; however, one of the unique PTMs of H3.3 is phosphorylation of serine 31 (S31ph), which has previously been linked to processes like mitosis (Hake et al., 2005). This unique residue in H3.3 aids transcription through different mechanisms. Martire et al. (2019) observed that H3.3 is enriched at enhancers of mouse embryonic stem cells (ESCs) and that the S31ph stimulates enhancer acetylation by histone acetyltransferase p300, which in turn promotes transcription. At the transcription start site (TSS) of active genes, S31ph enables rapid transcription, possibly by allowing easier access to the transcriptional machinery (Armache et al., 2020). A third study observed that when H3.3 is present at gene bodies, it promotes a stronger interaction of Zinc Finger MYND-Type Containing 11 (ZMYND11), an elongation corepressor, with H3K36me3, a transcriptional elongation histone PTM (Wen et al., 2014). This association is influenced by the presence of S31. However, when S31 is phosphorylated, the recognition of H3K36me3 by ZMYND11 is abolished since the corepressor cannot longer bind to the chromatin (Guo et al., 2014), suggesting that S31ph contributes to elongation of transcribed genes. Taken together, these studies nicely highlight the importance of a single amino acid, unique to a variant, in the regulation of transcription.

Another histone variant that has been broadly linked to transcriptional regulation is H2A.Z. This histone was originally linked to transcriptional activation (Wong et al., 2007; Sutcliffe et al., 2009). Moreover, it has been demonstrated that both the presence and proximity of H2A.Z are highly important for transcriptional activation. H2A.Z knockdown (KD) causes a reduction of RNA pol II at TSS (Hardy et al., 2009), and not only its enrichment but also the proximity of H2A.Z nucleosomes to TSS affects gene expression. Bargaje et al. (2012) described this in mouse liver by generating a genome-wide nucleosome position map and complementing with chromatin immunoprecipitation assay with sequencing (ChIP-seq); the results demonstrated that the closer H2A.Z was to the TSS, the higher the RNA pol II occupancy. In fact, it was believed that TSS of active genes contained regions depleted from nucleosomes, previously known as “nucleosome free regions,” but Jin et al. (2009) demonstrated that these regions were enriched with H3.3–H2A.Z nucleosomes, forming highly unstable nucleosomes that enabled faster transcription initiation through their eviction. This highlights the importance of studying nucleosomes as a unit, where specific combinations can cause different outputs in the dynamics of chromatin. In addition, the differences between homotypic and heterotypic nucleosomes should be taken into consideration for this type of study.

A third variant involved in the transcription of genes in brain and testes is H2A.B. Soboleva et al. (2017) demonstrated, through ChIP-seq, that the mouse ortholog of H2A.B is enriched at TSS and at the beginning of the gene body (excluding the +1 nucleosome). Interestingly, they found that in these tissues H2A.B can associate with mRNA at intron-exon boundaries, which led them to propose a model where H2A.B.3 (H2A.B variant 3) replaces H2A.Z, and recruits splicing factors. This is followed by direct interaction of H2A.B.3 and the transcribing RNA, facilitating the splicing by releasing the splicing factors. A previous study in HeLa cells stably expressing a plasmid with H2A.B found something similar: the variant is enriched at the gene bodies of active genes, it interacts with components of the spliceosome and RNA pol II, and it is involved in mRNA splicing (Tolstorukov et al., 2012). A third study in mouse ESCs uncovered the association of this variant with methylated DNA in gene bodies of certain imprinted loci. Although they did not experimentally describe the mechanism, they hypothesize that its presence at these hypermethylated regions facilitates transcription elongation by relaxing the chromatin, allowing the transcription machinery to surpass DNA methylation (Chen et al., 2014). In sum, due to the relevance of H3.3 and H2A.B in transcription regulation, it is important to further characterize if both H2A.B and H3.3 can work together during transcription and alternative splicing and if this is dependent on cell types. Cell-type dependency is particularly relevant considering that only a few tissues overexpress certain variants, and there could be tissue-specific replication uncoupled histones with a similar function or mechanism.

### Distinct histone variants are recruited to damaged DNA sites

Cells are constantly exposed to DNA damage insults, and in order to counteract them, they have developed different DNA damage response (DDR) pathways. Importantly, distinct histone variants and their chaperones are implicated in some of these pathways (Figure 1C). Once there is an insult, one of the early DNA damage markers in double strand breaks (DSB) is the phosphorylation on serine 139 of histone variant H2A.X (commonly known as γH2AX) (Rogakou et al., 1998). In ultraviolet-C radiation (UVC)-damaged cell lines, Piquet et al. (2018) demonstrated that the FACT complex deposits newly synthesized H2A.X to these damage repair sites, potentiating this chain-like mechanism of DNA damage signaling. Additionally, they found that while H2A.X is deposited, H2A.Z is evicted from these sites by ANP32E (acidic nuclear phosphoprotein 32 family member E), previously identified as a chaperone for this variant (Mao et al., 2014; Obri et al., 2014). In non-homologous end joining (NHEJ), it has been shown that the incorporation of this variant by ANP32E is important for the recruitment of Ku70 into the damaged region (Gursoy-Yuzugullu et al., 2015). It is worth mentioning that other remodeling chromatin factors apart from ANP32E are also related to the deposition of H2A.Z in DSBs, such as chromatin-remodeling ATPase INO80 and E1A-binding protein p400 (Xu et al., 2012; Alatwi and Downs, 2015; Begum et al., 2021). Nevertheless, the conditions and mechanisms implicated in their recruitment and activity still need further characterization.

It was previously reported that H3.3 can be deposited by HIRA at UVC-damaged chromatin (Adam et al., 2013), however it was still unclear which chaperone was in charge of this at heterochromatin regions. Fortuny et al. (2021) observed in mouse embryonic fibroblasts (MEFs), and further corroborated in MCF7 cells, that in pericentromeric heterochromatin the newly synthesized H3.3 is deposited by both HIRA (as the main driver) and, to a lesser and still unclear extent, the death domain-associated protein 6 (DAXX). By evaluating DNA damage with UVC, the authors proposed that HIRA is acting in the nucleotide excision repair (NER) pathway. However, further experiments involving different types of damage and DDR pathways are needed in order to contrast previous results and elucidate the importance of HIRA in these DNA reparation mechanisms. Compared to this, in telomeres which are also composed of heterochromatin, the deposition of H3.3 by DAXX and its partner the ATRX chromatin remodeler (ATRX) is important to maintain a correct p53 DNA damage response. The lack of either DAXX or ATRX in a glioblastoma cell line that has acquired an ALT (acquired lengthening of telomeres)-like phenotype, displays an accumulation of γH2AX at many p53 sites and, defects in p53 binding at key sites as well as in DNA damage response (Gulve et al., 2022).

Another H3 histone variant, whose role in DNA repair has not been clearly established, is CENPA (Zeitlin et al., 2009; Hédouin et al., 2017). Recently, Yilmaz et al. (2021) uncovered a novel and interesting mechanism by which, when DNA damage occurs during G1 phase, newly synthesized CENPA is deposited at the centromeric DSBs by HJURP (Holliday Junction Recognition Protein), reinforcing USP11 (ubiquitin carboxyl-terminal hydrolase 11) recruitment to enable RAD51 positioning at resected DNA ends. This allows homologous recombination (HR) to take place and maintain centromeric stability. This discovery enhances our understanding of the mechanisms employed by the centromere to face DNA damage, considering the important function of this region as well as its unique chromatin environment. Lastly, there is MacroH2A, an H2A variant which, besides being enriched on the inactive X chromosome (Costanzi and Pehrson, 1998) and facultative heterochromatin (Gamble et al., 2010), has been associated with different DDR pathways, as Caron et al. (2021) nicely cover in their review. For more detailed revisions on histone variants and chaperones involved in DNA damage and repair, please refer to Ferrand et al. (2020), Caron et al. (2021), and Chakraborty et al. (2021).

The role of histone variants and chaperones in replication, transcription, and DNA damage repair are the most common studied molecular mechanisms. However, recent findings about their importance in genomic stability and chromatin structure related to transposable elements is starting to unravel, and we discuss the findings regarding this topic in the next sections.

## Histone chaperones help in the regulation and silencing of transposable elements

Mobile genetic elements (MGEs) are any type of genetic material that can move within a genome or between cells (Leplae et al., 2004). The set of all MGEs in a cell constitutes its “mobilome,” and it can be divided into categories based on the mechanism of movement and the character of the mobile DNA sequences (Siefert, 2009). Examples of MGEs include plasmids, transposable elements (or transposons) and viral agents. Transposable elements (TEs) comprise around 40%–50% of mammalian genomes (Deininger et al., 2003). In comparison with other vertebrates where Class 2 (DNA) transposons govern, human and mouse genomes are dominated with Class 1 TEs, i.e., retrotransposons (Hellsten et al., 2010; van Kruijsbergen et al., 2017). Therefore, most studies on histone regulation of transposon activity in humans and mice have focused on retrotransposons. These can be subclassified into long terminal repeat (LTR) retrotransposons, such as the endogenous retroviruses (ERVs) superfamily and its members (ERV I, ERV II and ERV III families), and non-long terminal repeat (non-LTR) retrotransposons, such as the long and short interspersed nuclear elements (LINEs and SINEs, respectively) superfamilies (Bourque et al., 2018).

Transposons contribute to the variation, adaptation, and evolution of genomes through the duplication or deletion of genes or their regulatory elements (Lee et al., 2008; Longo et al., 2009; Feschotte and Gilbert, 2012; Sadic et al., 2015). TEs can also function as alternative promoters for neighboring genes, causing non-canonical regulation of transcription (Karimi et al., 2011; Rowe et al., 2013; Elsässer et al., 2015). Interestingly, transposons show a regulatory, developmental, and evolutionary relevance for mammalian genomes (Finnegan, 1989; Wright and Finnegan, 2001; Dodsworth et al., 2015; Jangam et al., 2017; Ricci et al., 2018; Rodriguez and Arkhipova, 2018). Nevertheless, their transcriptional, transpositional and retrotranspositional activity can also lead to detrimental genome instability, events of mutagenesis or cancerous transformations (Maksakova et al., 2008; Robberecht et al., 2013). Importantly, the transposons or viruses that enter or are activated, temporarily or permanently, in the nucleus often interact with the cellular chromatin machinery and can be subjected to the process of chromatin formation (Sultana et al., 2021). In the case of mammals, the major mechanism for TEs silencing is DNA methylation (Hatanaka et al., 2015). However, additional mechanisms are highly important to keep TEs silenced when demethylation processes occur, as in embryonic development, or when methylation systems fail, as in various pathologies. In this context, the participation of histone chaperones is critical to prevent the abnormal activation of retrotransposons, such as LINEs, SINEs and ERVs (Elsässer et al., 2015), as well as to regulate gene expression and maintain chromatin stability by depositing specific histone variants (Esteves de Lima et al., 2021; Viktorovskaya et al., 2021; Zhang et al., 2022). A list of transposable elements and the histone variants, heterochromatin-associated histone marks, and histone chaperones related to their regulation is shown in Supplementary Table S1. In the following sections, we will focus on transposons that interact with eukaryotes genomes, particularly those described as capable of causing detrimental effects on mammalian cells, and their regulation by histones and histone chaperones, particularly by DAXX, the chromatin assembly factor 1 (CAF-1), HIRA and FACT.

### CAF-1-mediated transposon regulation

**FIGURE 2:**
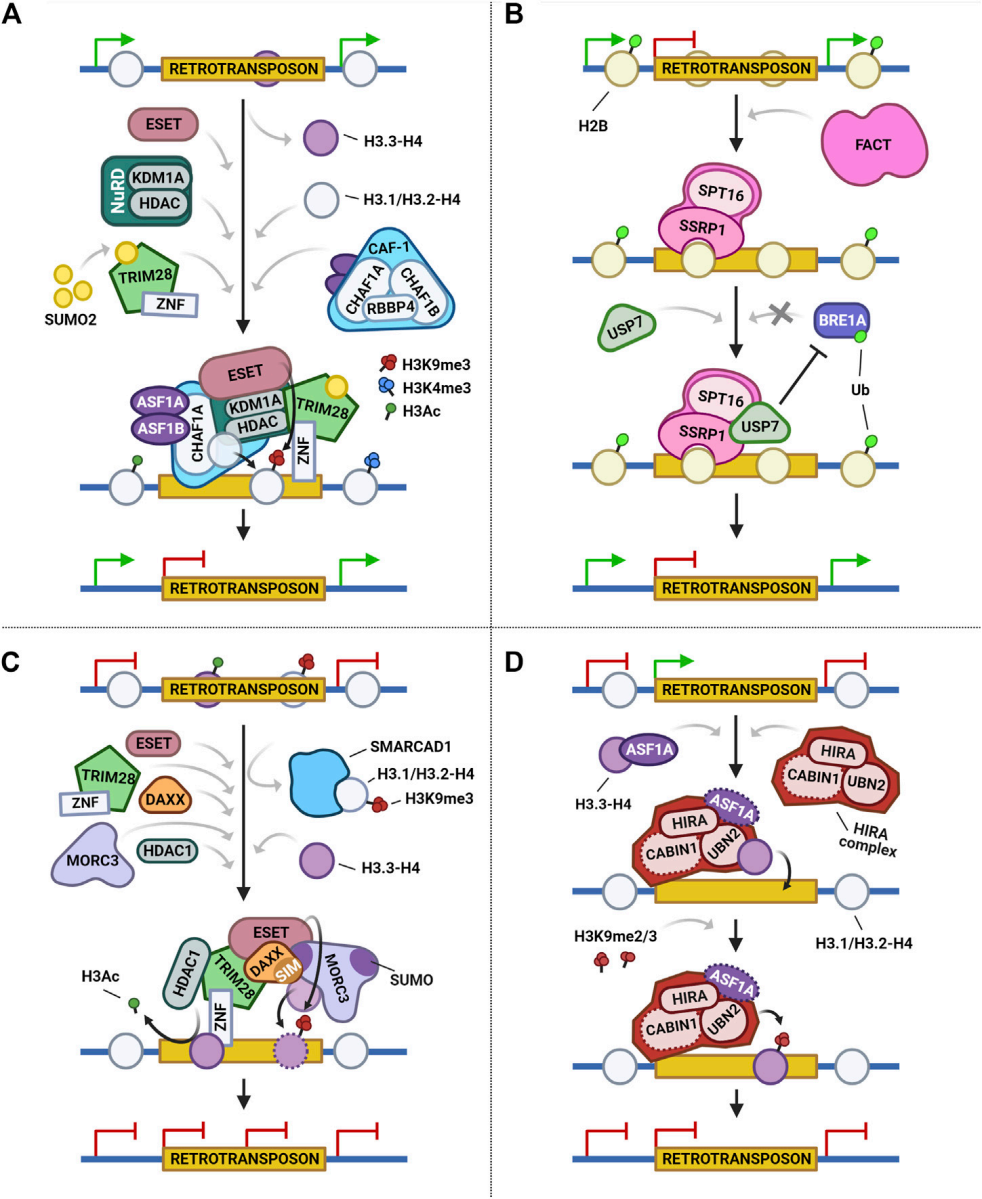
Four main histone chaperones show diverse and dynamic mechanisms for the repression of retrotransposon activity. **(A)** During retrotransposon regulation, the CAF-1 complex and its histone chaperones ASF1A/B promote the deposition of histone dimer H3.1/H3.2-H4 to mark integrated proviral retrotransposons. Localized transcriptional repression is then reinforced by members of the NuRD complex and ESET, which results in reduced H3K4me3 and H3Ac marks. SUMO2 sumoylates TRIM28, which is then recruited onto the proviral DNA and promotes the deposition of the repressive H3K9me3 mark. **(B)** The SRRP1 subunit of the FACT complex binds to retrotransposons and interacts with the SPT16 subunit and H2B deubiquitinase USP7. USP7 suppresses BRE1A, and decreases H2BK120ub deposition, thus repressing the expression of retrotransposons. **(C)** In DAXX-mediated retrotransposon regulation, TRIM28 is first recruited by KRAB-ZFPs (ZNF) and orchestrates heterochromatin formation and maintenance by recruiting ESET, DAXX, and SMARCAD1. SMARCAD1 activity leads to the eviction of existing H3.1/H3.2-H4 dimers. Subsequently, DAXX and MORC3 deposit H3.3-H4 and replace the lost histones. Finally, the complex promotes HDAC activity and ESET incorporates the H3.3K9me3 mark, which represses the retrotransposon. **(D)** The HIRA complex suppresses retrotransposon activity by interacting with and mediating the placement of H3.3 and its associated H3K9 methylation. Dotted lines represent interactors or activities reported to be dispensable for retrotransposon repression.

The CAF-1 complex is an H3-H4 chaperone conserved among all eukaryotes. It has important roles during DNA replication and repair, regulation of gene expression, and maintenance of chromatin accessibility (Liu et al., 2016). The CAF-1 complex is also part of the interconnected and complicated network of genetic and epigenetic mechanisms by which cells regulate retroviruses within genomes (Figure 2A). One example of this intricate mechanism is the restriction of the acquirement of 2-cell (2C) like state and repression of ERV III retrotransposons (muERV-L/MERVL: murine ERV-L elements; MaLRs: mammalian apparent LTR retrotransposons) in ESCs (Hatanaka et al., 2015; Ishiuchi et al., 2015; Yang et al., 2015). Additionally, hypomethylated preimplantation mouse embryos are protected from intracisternal A-particle retrotransposons (IAPs), LINE-1 and SINEB2, and show arrested development by repressive histone PTMs mediated by the CAF-1 complex (Hatanaka et al., 2015). To attain this, the CAF-1 complex mediates the replacement of H3.3 with H3.1/H3.2 on ERVs regions and the deposition of repressive histone marks, including H3K9me2, H3K27me3, and, predominantly, H3K9me3 and H4K20me3 (Hatanaka et al., 2015).

The CAF-1 complex has several interactors. Yang et al. (2015) reviewed the cellular factors involved in provirus repression in embryonic carcinomas (ECs) and ESCs, including subunits of the CAF-1 complex (CHAF1A/CHAF1B), sumoylation factors such as SUMO2 (small ubiquitin-related modifier 2), and chromatin modifiers, like TRIM28 (transcription intermediary factor 1-beta) and ESET (histone-lysine N-methyltransferase SETDB1). Their results demonstrated a recruitment of both CHAF1A and SUMO2 to deposit replication coupled histones H3.1/H3.2 on ERVs (MMLV: Moloney murine leukemia virus), as well as an increased repression *via* H3K9me3 and H4K20me3 marks. Specifically, CHAF1A reinforces transcriptional repression through its interaction with members of the NuRD complex (KDM1A: Lysine-specific histone demethylase 1A; HDAC1/HDAC2: Histone deacetylase 1 or 2) and ESET, while SUMO2 orchestrates the provirus repressive function of the canonical zinc finger (ZNF) protein 809 (ZFP809)–TRIM28–ESET machinery by sumoylation of TRIM28.

### FACT-mediated transposon regulation

Another important histone chaperone is the FACT complex, which mediates the deposition of H2A/H2B histones and binds to H3-H4 dimers simultaneously (Winkler and Luger, 2011; Wang et al., 2018; Chen F. et al., 2020). This complex is a stable heterodimer comprised of two multi-domain subunits, SSRP1 and SPT16 (Orphanides et al., 1999), and their ability to simultaneously engage with various histones makes the FACT complex unique among all histone chaperones (Zhou et al., 2020). Recently, Chen F. et al. (2020) hypothesized and subsequently demonstrated that the FACT complex is a suppressor of ERVs and ERV-driven cryptic transcription in ESCs (Figure 2B). In their study, loss of the SSRP1 component activated MERVL whereas the re-introduction of SSRP1 rescued the repression phenotype. Additionally, they observed that SSRP1 interacts with MERVL and suppresses cryptic transcription of MERVL-fused genes. Remarkably, SSRP1 also associates with and recruits epigenetic regulators, such as ubiquitin specific peptidase 7 (USP7), a known H2B deubiquitinase. USP7 acts by deubiquitinating H2BK120ub and thereby represses the expression of MERVL-fused genes. SPT16 also interacts with USP7; however, an intact FACT complex with an active SSRP1 subunit is needed for the effective recruitment of USP7 to MERVL (Chen F. et al., 2020).

Altogether, these results supported the mechanism that the SSRP1 subunit of the FACT complex recruits USP7 to repress MERVL and MERVL-fused genes in ESCs by impeding the ubiquitination of H2Bub. However, it is possible that other FACT-associated proteins may contribute to the repression of MERVL and MERVL-fused genes or that other pathways repressing cryptic transcription initiation may be regulated by the FACT complex itself (Chen F. et al., 2020).

### DAXX-mediated transposon regulation

As we addressed previously, the histone variant H3.3 can be incorporated at distinct regions of the chromatin by either the HIRA or ATRX–DAXX histone chaperone complexes (Drané et al., 2010; Goldberg et al., 2010; Lewis et al., 2010). While HIRA is responsible for H3.3 enrichment at genic regions, the ATRX–DAXX complex facilitates H3.3 deposition at heterochromatic and simple repeat regions such as telomeres (Wong et al., 2009; Goldberg et al., 2010; Lewis et al., 2010; Elsässer et al., 2015). Besides having several other roles coincidentally with its many modifiers (Dyer et al., 2017), DAXX has been found to deposit histone H3.3 to regulate retrotransposons in both mouse and human (Elsässer et al., 2015; He et al., 2015; Wolf et al., 2017; Wasylishen et al., 2020; Groh et al., 2021).

In this context, two models have been proposed for the mechanism by which DAXX and its interactors repress retrotransposons. Both models rely on the fact that ERVs are silenced through H3K9me3 by ESET (Matsui et al., 2010) and TRIM28 (Rowe et al., 2010, 2013); however, they differ in their involvement with ATRX and other interactors.

On the one side, a study by Elsässer et al. (2015) using ChIP-seq revealed that both DAXX and ATRX co-occupy ERVs (ERV I and ERV II) enriched with TRIM28 and ESET. Given their results, they suggested that 1) the recruitment of DAXX, H3.3 and TRIM28 to ERVs is co-dependent and occurs upstream of ESET, linking H3.3 to ERV-associated H3K9me3, and 2) the deposition at a subset of these TEs is dependent upon both ATRX and DAXX. Additionally, they reported that ATRX/DAXX deletion attenuates H3.3 enrichment at IAP ERVs, indicating that ATRX/DAXX is required for H3.3 enrichment at specific subclasses of ERVs (Elsässer et al., 2015). This model coincides with previous studies that reported that incorporation of H3.3 at silent genomic loci depend on ATRX/DAXX (Drané et al., 2010; Goldberg et al., 2010; Lewis et al., 2010).

On the contrary, a structural and biochemical study by Hoelper et al. (2017) led to the identification of two functionally and mechanistically distinct DAXX–H3.3–H4-containing complexes involved in the maintenance of repressed chromatin states. One complex corresponds to the first model mentioned. It is in fact the second complex, which contains DAXX, histones H3.3–H4, ESET, TRIM28, and HDAC activity, the one that helps facilitate the repression of ERVs in mouse ESCs (Figure 2C). Certainly, these data suggest that ATRX and ESET-TRIM28 have mutually exclusive nature, and that the deposition of histone H3.3 at ERVs is dispensable for DAXX–ESET–TRIM28-mediated repression.

The set of elements of the DAXX-mediated transposon regulation mechanism is continuously being elucidated. Recently, Groh et al. (2021) found that the ATPase cycle and the sumoylation of the MORC family CW-type zinc finger protein 3 (MORC3) are necessary steps for DAXX’s ERV-chromatin regulation, as DAXX needs to interact with the sumoylated version of MORC3 through its SUMO interaction motif to be able to contribute H3.3–H4 dimers. Thus, this data reveals yet another critical regulator of DAXX-mediated histone H3.3 incorporation to ERV regions.

### HIRA-mediated transposon regulation

The specific activity of HIRA lies in its ability to recruit and form complexes with histone-modifying proteins such as histone acetyltransferases and HDACs and to help regulate specific histone variant depositions (Ray-Gallet et al., 2002; Tagami et al., 2004; De Koning et al., 2007). In general, the HIRA complex (composed of HIRA protein, UBN1 or UBN2 and calcineurin-binding protein CABIN1) suppresses retrotransposons by mediating the deposition of histone H3.3-H4 onto chromatin independently of DNA synthesis (Figure 2D) and limiting the generation of retrotransposon-derived long non-coding RNAs (lncRNAs) (Zhang et al., 2022). However, the subunits of the HIRA complex act distinctly in silencing retrotransposons (Macfarlan et al., 2012; Zhang et al., 2022): the HIRA subunit mainly recognizes and suppresses ERV I retrotransposons (such as RLTR4, RLTR12H and RLTR1B) and ERV II retrotransposons (such as ERVB4_1B, IAPLTR3-int and IAPLTR2b) through H3.3-H4 deposition. In contrast, the UBN2 subunit mainly represses LINE-1 and ERV III retrotransposons (such as MERVL, MMERGLN_LTR and MTA) not only through H3.3 deposition, but also through the installment of H3K9me2 and H3K9me3 marks. The role of the UBN1 subunit in repressing retrotransposons is significantly weaker and specific to certain ERVs subfamilies, such as RLTR4 and MERVL. The results of Zhang et al. (2022) also suggest that HIRA, UBN1, and UBN2 have specific roles in silencing the expression of TEs and other genes; in other words, HIRA and UBN1/UBN2 show high specificity in recognizing different classes of retrotransposons.

For the proper functioning of cellular processes, fine-tuning of the chromatin must be assured through the previously described mechanisms and genomic components that involve a balance of histones and their chaperones. Nevertheless, in health and disease the balance and function of the histones and chaperones changes, as we discuss in the following sections. Aging is a natural process, where recently the role of these epigenetic components is being elucidated. On the other hand, diseases, such as cancer, alterations, and mutations in both histones and their chaperones have been described. We will not review the information on cancer, but we invite the reader to refer to the following literature on the topic: Nye et al. (2018), Nacev et al. (2019), and Ghiraldini et al. (2021). Nonetheless, we will briefly discuss what is known about syndromes where histone chaperones are severely affected.

## Histones, their chaperones, and their involvement in aging

**FIGURE 3:**
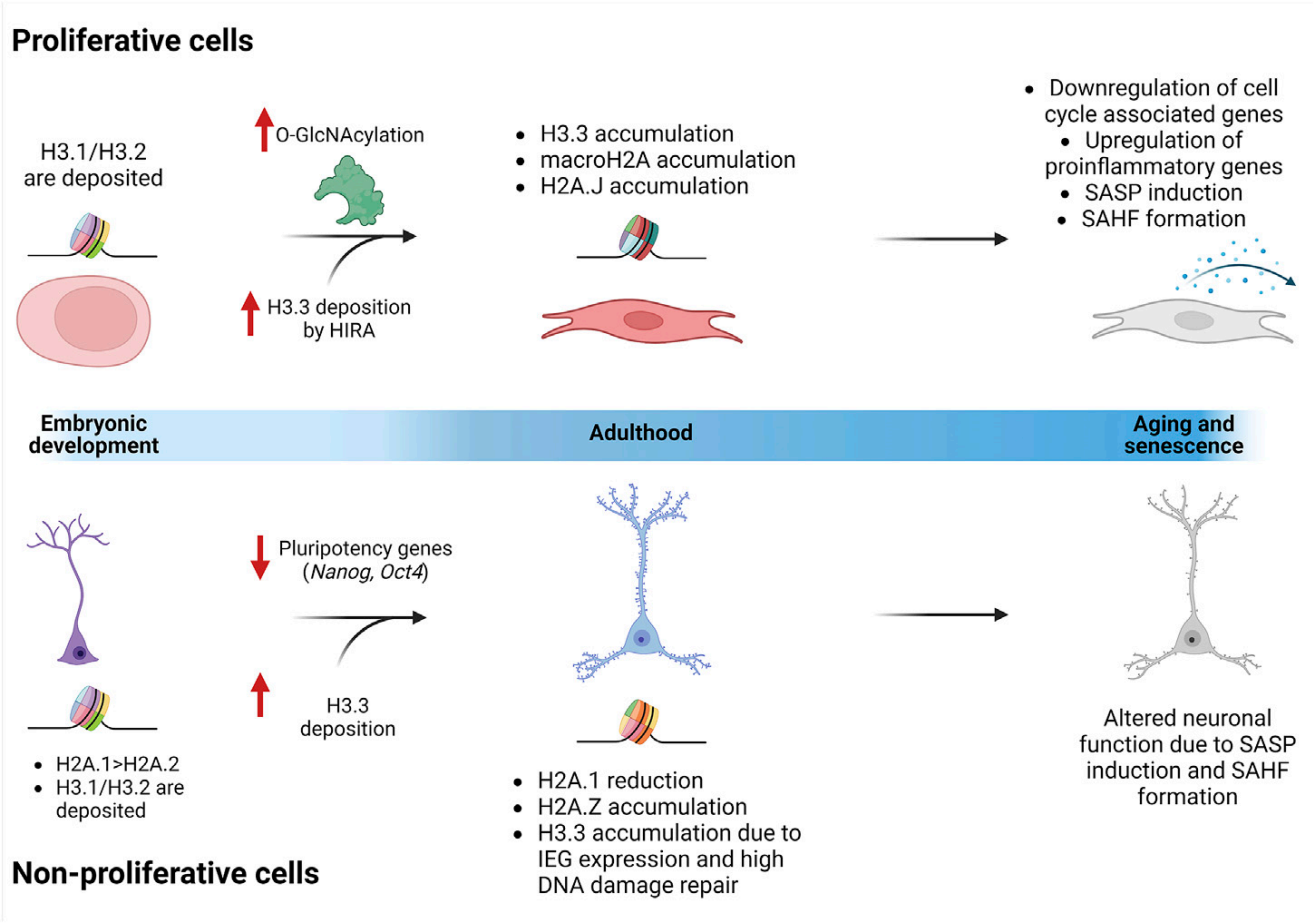
Replication uncoupled histones accumulate through life differently in proliferative cells and non-proliferative cells. During embryonic development, H3.1 and H3.2 are deposited in the chromatin of all cells. In certain cell types, like neurons, some histone variants are enriched. For example, there is a higher amount of H2A.1 than H2A.2. In proliferative cells, O-GlcNAcylation has been described as an important PTM that is enriched as the cells age. Chaperones, like HIRA, increase their activity when O-GlcNAcylated, generating an accumulation of H3.3. In neurons, it has been speculated that H3.3 accumulates and substitutes H3.1/H3.2 due to the activation of IEGs and the constant DNA damage repair mechanisms. Additionally, different H2A variants are accumulated in proliferative cells (macroH2A and H2A.J) and in non-proliferative cells (H2A.Z) through the aging process. All these nucleosome changes are involved in the activation of the senescent program, altering tissue-specific functions.

Aging is a life-lasting event that results from the accumulation of damage and different molecular and cellular alterations through time, leading to a deterioration of physiological functions that are necessary for survival. Epigenetic alterations are a major contributor to cellular senescence, including the exchange of replication coupled histones for variants, and their concomitant accumulation. Given this, it is important to address the latest findings about the role of histone variants in cellular senescence and how they can contribute to altered gene expression and overall aging in proliferative vs. non-proliferative cells (Figure 3).

### Aging in proliferative cells: Cellular senescence in retrograde

Cellular senescence can be defined as a permanent or highly stable state of cell cycle arrest (Maciel-Barón et al., 2018). In proliferative cells, senescence aims to lower the replication of damaged and old cells that have accumulated molecular alterations. It is a physiological process that happens throughout the life of an organism, in certain moments such as embryogenesis and tissue remodeling (Hernandez-Segura et al., 2018; Herranz and Gil, 2018; Calcinotto et al., 2019) or as a protection mechanism to suppress malignant transformation and proliferation (Rai and Adams, 2012). The senescent program might be induced due to different types of stressors, such as oxidative stress, radiation, autophagy impairment, among others; this is known as premature senescence.

As mentioned, senescence involves changes at molecular and cellular level, which can be related to organelle function, gene expression, epigenetic regulation, disruption in the energy metabolism, etc. Given this, several biomarkers have been established to characterize the senescent phenotype. A senescence-associated secretory phenotype (SASP) of several proinflammatory molecules, like cytokines and some other molecules, such as growth factors and proteases, is seen (Acosta et al., 2008; Coppé et al., 2008). Besides the molecules involved in the SASP, the detection of the senescence-associated β-galactosidase (SA-β-Gal), the presence of γH2AX nuclear foci, increased heterochromatin foci (known as senescence-associated heterochromatin foci—SAHF) and the expression of macroH2A are also used as biomarkers to identify a senescent cell (Bayreuther et al., 1988; Dimri et al., 1995; Rodier et al., 2011; Moreno-Blas et al., 2018).

It is interesting to highlight that there are numerous epigenetic alterations seen as important elements of the senescent phenotype. Yet, only a few histone variants, such as macroH2A and γH2AX, have been studied, while others, like H3.3, have started to become widely explored, particularly because of their role in chromatin regulation and gene expression.

In an adult organism, proliferative cells are expected to accumulate H3.3 in their chromatin due to the replication-independent kinetic deposition of the variant compared to the canonical H3.1/H3.2 histones. Some authors hypothesize that the accumulation of H3.3 in certain regions can be a strategy of the cell to follow a semiconservative replication and to “hold/mark” the sites where H3.1/H3.2 need to be deposited once the cell replicates; since eventually these cells stop replicating, H3.3 stays in those regions substituting the canonical histone (Saade et al., 2015). This H3.3 enrichment was corroborated in the liver, kidney, brain, and heart of aged mice vs. young mice (Tvardovskiy et al., 2017). Additionally, overexpression of H3.3 but not H3.1 histone variant induces senescence in fibroblasts (Duarte et al., 2014). As seen by Tvardovskiy et al. (2017), not only terminally differentiated cell types accumulate H3.3, but also slow-dividing mitotic tissues, like the kidney and liver. This highlights the importance of H3.3 in aging tissues, independently of their mitotic rate.

The PTMs in canonical or histone variants add another level of complexity to the mechanisms of H3.3 accumulation through the aging process. Enzymes in charge of establishing PTMs selectively identify protein sequences, thus affecting the outcome: the functional readout. In this sense, the ZMYND11 protein specifically recognizes H3.3K36me3 in the body of genes where it regulates the elongation rate of the RNA pol II (Wen et al., 2014). Additionally, tissues like the heart, liver, and kidney show an increment of H3.3K36me2, while H3.3K9me2 decreases through aging. Interestingly, some PTMs, like H3K27me2/me3, are only altered in specific tissues, such as the liver or the heart (Tvardovskiy et al., 2017). These data suggest that alongside H3.3 accumulation in aging organisms, there is an alteration in methylation marks that will affect the “meaning” of the histone code and the biological outcome.

Chromatin reorganization is an important feature of senescent cells. When seen under a microscope with 4′-6-diamino-2-phenylindole (DAPI) staining, senescent cells show punctate DNA foci instead of the characteristic heterogeneous distribution of the staining that proliferating cells have. These are the SAHF, and they are enriched with macroH2A. The deposition of this variant is dependent on the HUCA histone chaperone complex activity (Zhang et al., 2005), the same remodeling complex that can deposit H3.3, thus suggesting the involvement of the H3.3 histone variant in the formation of the SAHF.

Another important characteristic of the H3.3 accumulation in aging cells is its proteolytic cleavage which leads to the presence of H3.3cs1. Fibroblast genome-wide transcriptional profiling shows that this cleavage product is enough to induce the senescent program by silencing cell cycle regulators and RB/E2F target genes (Duarte et al., 2014). Authors suggest that the proteolytic cleavage of H3.3, diminishes the H3K4me3 in the regulatory regions of these genes, leading them to a permanent repressed state. Additionally, another variant implicated in the induction of the senescent phenotype is the H2A.J. This variant accumulates when the DNA of the senescent cells is excessively damaged and gets enriched at specific sets of genes associated with the SASP, such as *IL1B*, *CCL2*, and *CXCL5*, as seen in aged tissues like the liver, kidney, and brain (Contrepois et al., 2017).

Furthermore, HIRA’s activity can be modulated by different PTMs like the O-linked N-acetylglucosamination (O-GlcNAcylation). This PTM is an important regulator of the HIRA complex: when absent, H3.3 deposition is reduced (Lee and Zhang, 2016). Interestingly, several studies show that alterations in the O-GlcNacylation of proteins is increased in aged tissue (Fülöp et al., 2008), suggesting that the increase in HIRA’s O-GlcNacylation might potentiate H3.3 deposition promoting cellular senescence. The described mechanisms are summarized in Figure 3.

Most of the works and reviews show evidence of how epigenetic alterations are related to senescence through the perspective of cellular replication. Meaning, most of them focus on available reports on proliferative cells, such as fibroblasts (Maciel-Barón et al., 2018). Nevertheless, it is important to address how the epigenetic machinery changes through the aging of non-proliferative cells, like neurons, which can lead to pathological conditions.

### Aging in non-proliferative cells: Neurons at a glance

During the neurogenic process in embryogenesis, there is a differential deposition of histone variants. H2A.1 is enriched twice as much as H2A.2 both in neurons and neuroblasts. Nevertheless, during postnatal development H2A.1 decreases, suggesting that H2A.2 has more stability. On the other hand, another H2A variant, H2A.Z presumably deposited by the INO80 subfamily chromatin remodelers (Papamichos-Chronakis et al., 2011; Mossink et al., 2021), accumulates in aging neurons, pointing out a possible implication in neuronal mechanisms (Stefanelli et al., 2018). Regarding H3 histone variants, neuroblasts show enrichment of the canonical H3.1 and H3.2 which are later replaced by H3.3 in mature neurons (Piña and Suau, 1987; Bosch and Suau, 1995). Interestingly, as neurons age, H3.1 has a slow turnover with only 10% of the protein being replaced every 6 months, allowing H3.3 to increase its levels, replacing almost all the canonical histone variants, and accumulating through aging (Maze et al., 2015). Given this, the H3.1 found in the adult brain corresponds to the ones deposited at a young developing age.

The deposition of H3 variants changes in a gene-dependent manner. Experiments made with ESCs differentiated to neuronal precursors (NPC) showed that when the expression of pluripotency genes decreases, such as *Nanog* and *Oct4*, the H3.3 enriched in the gene bodies is lost (Goldberg et al., 2010); while tissue-specific body genes are enriched with this variant (Banaszynski et al., 2013; Maze et al., 2015). Furthermore, in bivalent genes that evolve to become transcriptionally active, H3.3 is kept around the TSS and is incorporated into the gene body and in those that become transcriptionally inactive, the H3.3 is lost around the TSS (Goldberg et al., 2010). Additionally, haploinsufficiency of HIRA leads to abnormal defects involved in the regulation of neuronal differentiation and maturation (Jeanne et al., 2021). This places histone variants and their chaperones as important regulators of neuronal development and maturation.

In mature cortical neurons, activity-dependent modifications of chromatin contribute to changes in their circuitry by activating genes such as *Bdnf Exon IV*, *c-Fos*, *Dusp6*, among others. These genes, which are known as immediate early genes (IEGs) because they are transiently and rapidly activated upon neuronal activation, need a rapid chromatin remodeling, in part mediated by DAXX, which loads H3.3 at their regulatory elements (Michod et al., 2012), while other mechanisms are responsible for evicting H2A.Z at the TSS-flaking sites (Zovkic et al., 2014; Dunn et al., 2017; Stefanelli et al., 2018). Upon DAXX silencing, depolarization of neurons could not increase H3.3 in the regulatory regions and led to a diminished mRNA transcription of these IEGs. Interestingly, H3.3 KD experiments made in neuronal cultures show a reduction of dendritic spines and in the adult mice the miniature excitatory postsynaptic currents, that reflect synaptic maintenance, were diminished (Maze et al., 2015). Furthermore, H3.3 KD experiments made in primary astrocytic cultures showed that, even after adding H3.3 again, the gene-specific regulation was recovered (Maze et al., 2015). These experiments suggest that in proliferating cells, like astrocytes, other H3 variants (H3.1 or H3.2) can rescue the function; while in neurons the H3.3 variant is extremely important to regulate neuronal activity and circuitry.

Compared to H3.3, H2A.Z, though it is enriched in aging neurons and remains responsive, serves as a negative regulator of gene expression associated with learning and memory (Zovkic et al., 2014). Interestingly, H2A.Z dependent-memory formation seems to be sex-specific since it has a higher binding in female vs. male mice and the conditional knock-out (KO) enhanced fear memory in male, but not in female mice (Ramzan et al., 2020). These experiments strongly suggest that, even though H3.3 and H2A.Z accumulate in neuronal chromatin altering the nucleosome composition, both remain highly dynamic to regulate the non-proliferating cell type-specific gene expression associated to neuronal plasticity and cognition.

When using transgenic mice harboring an H3.3-HA tagged variant, overexpression of this histone variant leads to an impaired contextual fear memory and motor learning (McNally et al., 2016). Furthermore, in a mouse model of depression under a chronic stress paradigm, the quantification of one of the two genes encoding H3.3, *H3F3B*, shows an upregulation of the mRNA altering transcriptional programs (Lepack et al., 2016), possibly associated with aberrant synaptic plasticity. The *H3F3B* gene overexpression was also seen in the nucleus accumbens (NAc) of human postmortem brains diagnosed with major depressive disorder (Lepack et al., 2016). On the contrary, mice exposed to an enriched environment show an increased H3.3 turnover, suggesting that environmental stimuli are implicated in *H3F3B* transcription and its H3.3-encoded variant deposition (Maze et al., 2015). Additionally, H3.3 was found to be enriched in the genomic regulatory regions of four genes associated with chronic cocaine consumption in the NAc in mice (Wimmer et al., 2019), further confirming the importance of H3.3 in barcoding transcriptional sites. Although additional experimental data is necessary to prove why particular genes and regulatory regions are affected, these studies suggest that accumulation of H3.3 may have detrimental effects on specific brain structures that are associated with the performance of certain behavioral tasks or with the development of neuropsychiatric disorders and addictions.

Furthermore, since non-proliferative cells, like neurons, will no longer divide and replicate their DNA, the substitution of the H3.3 variant deposited during DNA damage repair by HIRA (Adam et al., 2013; Frey et al., 2014) by the replication dependent canonical H3 is not possible. This can be another reason H3.3 accumulates in neuronal chromatin throughout life. Even though senescent neurons maintain highly dynamic chromatin through the deposition of newly synthesized histone variants, its accumulation might be a reason the neuronal transcriptional program starts to fail as the organism ages. Figure 3 summarizes some of the changes described previously and characterized up until now in neurons.

Histones and their chaperones are naturally affected through aging, and the organism finds a way to surpass these changes. Nevertheless, inborn errors in these mechanisms have been characterized, and are important to address in the following section.

## Chromatin remodelers and histone chaperones as targets in syndromes

Only a few so-called “chromatin remodeling syndromes” and pathologies where the function of the chaperones is compromised (“associated-like” syndromes) have been described (Table 2). These are syndromes that harbor alterations affecting histone variants, their chaperones and chromatin remodelers. Furthermore, probably due to the lack of proper technology to study the variants and chaperones involved in the syndromes, the information is scarce. However, there is evidence worth mentioning and highlighting in the next sections.

**TABLE 2:** General aspects of known chromatin-remodeling and associated-like syndromes.

Syndrome	Altered histone/chaperone	Gene	Altered mechanism	References
Di-George syndrome	H3.3/HIRA	*HIRA*	Restriction to chromatin	Chen et al. (2020a)
Fanconi Anemia-ATRX	H3.3/DAXX	*FANCD2, ATRX*	Chaperone activity, promotion of replication forks	Dyer et al. (2017)
Kabuki syndrome	H3	*KMT2D, KDM6A*	Histone methylation and demethylation	Wang et al. (2019)
Rett syndrome	H3	*MECP2*	Hyperacetylation	Amir et al. (1999)
Rubinstein–Taybi syndrome	H3	*CREBBP*	Histone acetylation	Murata et al. (2001)
Coffin–Lowry syndrome	H3	*RSK2*	Histone phosphorylation	Delaunoy et al. (2001)

Although the information is limited, it is known that alterations indirectly affecting histones can be an important characteristic of some known syndromes. This is because the deposition of PTMs is compromised due to mutations in enzymes in charge of this activity. For example, Kabuki syndrome is caused by a mutation in KMT2D, a methyltransferase of lysine 4 (K4) histone H3 (H3K4), leading to reduced histone methylation (Boniel et al., 2021). Another example are the mutations in the CREB binding protein, an H3 acetyltransferase altered in Rubinstein-Taybi syndrome (Attar and Kurdistani, 2017); or in the *rsk-2* gene in Coffin-Lowry syndrome, affecting phosphorylation of histone H3 (Ausió et al., 2003). As mentioned, these are not histone or chaperones alterations *per se*; however, they all end up altering the epigenetic histone landscape, leading to an impaired function of these proteins. Additionally, these affections may end up modifying not only marks on H3, but also on its variants. This phenomenon was described in pediatric glioblastoma, where a reduction in the H3K27me3 might reflect an alteration in the deposition of H3.3K27me3, which has been proposed as a dominant-negative effect of the mutant H3.3 (Bender et al., 2013). This suggests that similar mechanisms might happen in some syndromes that we are classifying as “chromatin remodeling associated-like syndromes.” Additionally, there are some other syndromes that are emerging research areas in the histone chaperone field. For example, for the H2A variants, mutations of *LSH/HELLS* gene, a chromatin remodeler known to interact with MacroH2A, causes Immunodeficiency Centromeric Instability Facial Anomalies (ICF) 4 syndrome, characterized by immunodeficiency, neurologic defects, and reduced growth (Xu et al., 2021). Therefore, histone variants and their chaperones are a potential field of study to understand their role in gene regulation and their involvement in pathologies.

### DiGeorge syndrome

DiGeorge syndrome, also known as 22q11.2 deletion syndrome, is the most common chromosomal microdeletion disorder (McDonald-McGinn et al., 2015) in which a 5-3 megabase (Mb) portion is deleted. Among the genes lost in this deleted region, *HIRA* is included. As it was previously mentioned, HIRA chaperone, when forming a four-subunit complex, is in charge of the deposition of H3.3 in many regions of the genome; for example, DNA damage sites and also bivalent genes in ESCs. For hematopoietic stem cells (HSCs) to function properly, epigenetic regulation needs to be carried out, including chromatin remodeling and histone PTMs. Therefore, aberrant epigenetic modifications may lead to impaired HSC development (Chen C. et al., 2020).

Even though HIRA is described as an important contributor for hematopoietic development, its whole function and role has not been completely elucidated. However, HIRA KO experiments cause a massive loss of bone marrow HSCs, derailing the generation of the hematopoietic lineage. Additionally, through the assay for transposase-accessible chromatin with sequencing (ATAC-seq), it was proven that HIRA KO restricts the access to chromatin affecting regions crucial for the transcription of HSC-specific genes. *Meis*, *Mecom*, *Fos*, *Jun*, and *Hoxa9* are among the genes that were found to be downregulated in the absence of HIRA, and some of them play a key role in HSCs development. For this reason, it is known that HIRA is necessary for the opening of chromatin sites to maintain the correct development of bone marrow HSCs, as well as a proper transcription of their genes (Chen C. et al., 2020).

The phenotype seen in these patients is very heterogeneous, including heart defects, parathyroid hypoplasia, immune deficiency, and hypocalcemia (Jeanne et al., 2021). Given this, the altered mechanisms that lead to its heterogeneous pathogenesis might depend on the cellular type analyzed, whether it is a neuronal or a hematopoietic stem cell defect. Currently, only a few authors have addressed alterations on histone variants as an etiology (Roberts et al., 1997; Farrell et al., 1999; D’Antoni et al., 2004). Further investigation is required to completely understand the whole landscape of this syndrome, especially the full implications of the absence of HIRA in this disorder.

### Alpha-thalassemia X-linked intellectual disability syndrome and Fanconi anemia

ATRX is a chromatin remodeler named after the developmental syndrome on which it was found mutated, the a-thalassemia/intellectual disability syndrome, X-linked (Dyer et al., 2017). Through its N-terminal PHD/Zinc finger domain, which consists of a GATA-like zinc finger and a PHD finger, the last one shared with the DNA methyltransferases DNMT3A, DNMT3B, and DNMT3L; ATRX recognizes H3K9me/me2/me3. As methyl groups are added to H3K9, the binding affinity between histone H3 and ATRX increases (Dash et al., 2018). Therefore, when this region is mutated, this property is affected, meaning that accessibility of several DNA methyltransferases to chromatin is lost (Ausió et al., 2003). Besides forming a complex with SWI/SNF2, whose role has been well characterized as a chromatin remodeling complex, ATRX associates with DAXX, and they are in charge of H3.3 deposition. Given this, any alteration in ATRX will affect this replication-uncoupled histone chromatin incorporation, which is the reason this syndrome is considered a chromatin remodeling syndrome. Fanconi anemia (FA) is a chromosome instability syndrome (Dyer et al., 2017) which occurs following germline mutations that result in high predisposition to cancer (Nepal et al., 2017). The FA core complex is constituted of eight proteins, and it is recruited to chromatin during replication fork stalling by FANCM. FANCD2 is the central protein in the FA pathway, which is responsible for genome stability during DNA replication (Chaudhury et al., 2013). Recently, new functions of ATRX were described, such as a physical and functional interactor with FANCD2, promoting its stability, and also as a protector of hydroxyurea (HU)-stalled replication forks and the promotion of replication fork restart. This led to question if DAXX along with ATRX, could associate with this newly described complex and what role it might have here.

With a DNA fiber analysis after HU-mediated replication fork stalling, one study evaluated the proportion of restart-competent replication forks between DAXX, FANCD2 and DAXX/FANCD2 double-deficient cells and found out they were similarly reduced. These results suggest that, when replication stress occurs, FANCD2 is ubiquitinated and locates to stalled replication forks to recruit homologous recombination factors and promote a replication fork start, while suppressing new replication origins; cooperating with the ATRX/DAXX complex, most likely including its H3.3 chaperone activity (Dyer et al., 2017).

## Challenges in the approaches to study histones and their chaperones

The study of histones, their variants, and their role in different cellular processes has been challenging due to the complex nature of these proteins. For instance, at the protein level they can share a high degree of similarity, being different in a few amino acids and at the gene level in most organisms their genes are encoded in multiple copies arranged in clusters. The use of traditional KO and KD technologies to study the effect of their absence is challenging not only for their gene distribution and mRNA stability, but also because of their relevance in several biological processes, as reviewed here. An ingenious way to come around this is to downregulate the expression of key elements involved in the unique biogenesis of the histone’s mRNA. One of these approaches includes the silencing of the Stem-loop binding protein (SLBP) (Sullivan et al., 2009). This protein binds the 3′ stem-loop mRNA end, it is involved in all the mRNA processing steps and its unique known target are these histones (Marzluff and Koreski, 2017). By generating a stable cell line that expressed an inducible small hairpin RNA (shRNA) to silence this factor, Jimeno-González et al. (2015) performed a genome-wide analysis on the effect of transcription when only low levels of canonical histones are available. The downside of this approach is that it is not able to distinguish between histones, therefore future targeted mechanisms could improve this if different components in mRNA processing are found within the core histones canonical mRNA. For example, in *Drosophila* it was found that the clusters of genes of the linker histone H1 and the core histones (H2A, H2B, H3, and H3) could be distinguished by the transcription factors that bind to the respective promoters. While TBP (TATA-box-binding protein)-related factor 2 (TRF2) regulated the H1 gene promoter, the core histones genes were controlled by TBP/TFIID (Isogai et al., 2007). This differential mechanism of transcriptional activation within the histone cluster, provides the first steps in targeted canonical histone specific silencing. However, to our knowledge, in mammals this is yet to be explored. Compared to replication-coupled histones, the genes encoding histone variants and histone chaperones are encoded by independent non-clustered genes, their expression is not limited to cell cycle regulation, and their mRNAs have 3′ polyA tails, therefore traditional KO and KD technologies are suitable for their study.

At the protein level, a collection of methods and techniques have evolved to study the interactions of histones and chaperones. For a detailed revision on this topic, based on H3 but applicable to all histones, please refer to Scott and Campos (2020). However, here we would like to highlight a few of the golden standard techniques still used nowadays for the study of histones and their chaperones, as well as one that has gained adepts in the field that allows the label and following histones *in vivo*: SNAP-tag System.

One of the first methods to isolate histones was through acid extraction of whole protein extract (Murray, 1966). This method relies on the nature of histones, since they are highly basic and do not precipitate easily under acid conditions. The most frequent acids employed for this are hydrochloric acid or sulfuric acid. However, some issues with this type of extraction are that for further analysis of the histones, pH must be neutralized, and certain PTMs are prone to be lost due to the acidity during the sample extraction. Another method that has efficiently extracted histones is the high-salt concentration method, where the composition of amino acids of the histones influences the strength of the bonds with DNA. The stronger the bond, the higher the concentration of salt required to dissociate the histones from the DNA. Therefore, this method, apart from extracting histones, can also function to measure the stability of the nucleosome composed by different histones and their variants. Shechter et al. (2007) detail standard protocols to isolate histones, as well as further purification techniques.

To study the deposition pattern of histones and their chaperones in chromatin, one of the gold standard methods is ChIP (Kuo and Allis, 1999). This technique allows us to determine the distribution and enrichment of proteins in genomic regions, in this case, histones or their chaperones. When coupled with sequencing, the distribution, biological effects, as well as chromatin states can be studied at a genome-wide level. Truch et al. (2018) provide a ChIP-seq protocol specially optimized for proteins that interact with chromatin through protein-protein interactions, for example, ATRX. One of the downsides of this technique is the requirement of specific antibodies to immunolabel histone variants and their chaperones. If we consider that most of the histone variants are understudied, the availability of commercial antibodies can become a barrier. For example, in the case of H2A.B although a couple of commercial antibodies are available, they have not been validated for ChIP. Given this, the one study that has evaluated H2A.B enrichment through ChIP-seq produced its own antibody (Soboleva et al., 2017). In addition, even when antibodies are available, specificity and affinity should also be taken into consideration, because the production of specific antibodies for a variant becomes an issue if the differences between histones are only a few amino acids, making the epitopes fairly indistinguishable from one another. In addition, usually it is necessary to use a high quantity of antibody per immunoprecipitation to properly immunodetect the protein of interest in the samples. This limits the number of assays that can be performed per antibody vial, raising the monetary costs per experiment. Another setback of ChIP is the quantity of the sample. Generally, it requires large amounts of cells or tissue, and when the original sample is limited, it becomes an issue. Nonetheless, there are improvements in the technique that allow the use of small quantities of the sample, lowering the input requirements (Dahl and Gilfillan, 2018). Nevertheless, the widespread usage of ChIP ranges around 1 × 10^7^ cells.

Nowadays, there are also several variations of the technique that grants different approaches, like the chromatin immunoprecipitation followed by selective isolation of chromatin-associated proteins (ChIP-SICAP). This ChIP-based technology allows differentiating stable from labile protein interactions and capture the stable ones using DNA biotinylation (Rafiee et al., 2016). Chromatin occupancy after replication with sequencing (ChOR-seq) and sister chromatids after replication with sequencing (SCAR-seq) may be employed for the study of replicating chromatin (Petryk et al., 2021), raising the possible techniques that can be used to study chromatin in different cellular contexts; in this case how histone variants and their chaperones are recruited and engage during S phase.

Finally, another technique that has been widely used to study histones and chaperones is the SNAP-tag system developed by Keppler et al. (2003) as a technique for labeling and tracking proteins. This technique uses a mutant DNA repair protein (O6-alkylguanine-DNA alkyltransferase) that binds to benzylguanine irreversibly. Benzylguanine can be coupled to different molecules, including fluorophores, allowing an *in vivo* tracking of newly synthesized proteins *via* a fluorescent microscope. Based on this method, the “quench-chase-pulse” assay was developed to follow and distinguish between new and old histones. Bodor et al. (2012), Clément et al. (2016), and Torné et al. (2018) provide protocols based on this technique focused on the study of histones as well as their chaperones. The development of this assay has drastically changed the way histones are studied. Allowing not only to differentiate between them, but also to follow the *in vivo* dynamics, order of deposition, chaperones involved, and the distribution pattern in the nucleus. All of which provide a better understanding of these essential proteins.

## Concluding remarks

During the last few years, the study of histone variants and their chaperones has increased considerably. However, most of these studies have focused on only a few variants and chaperones, resulting in scarce information. Aside from H3, H2A, and its variants, further studies addressing the rest of the histones are needed to have a bigger picture of how they influence chromatin regulation. Nevertheless, new emerging technologies are improving the experimental approaches for their study. Given this, we highlight the importance of further research about histone variants and their chaperones in the associated syndromes, physiological processes like aging, and their role in molecular processes, such as replication, transcription, DNA damage and their contribution to the regulation of transposable elements. This will further shed light on their involvement as important regulators of the ever-changing chromatin.

## References

[B1] Acosta J. C. O’Loghlen A. Banito A. Guijarro M. V. Augert A. Raguz S. (2008). Chemokine signaling via the CXCR2 receptor reinforces senescence. Cell 133, 1006–1018. 10.1016/j.cell.2008.03.038 18555777

[B2] Adam S. Polo S. E. Almouzni G. (2013). Transcription recovery after DNA damage requires chromatin priming by the H3.3 histone chaperone HIRA. Cell 155, 94–106. 10.1016/j.cell.2013.08.029 24074863

[B3] Alatwi H. E. Downs J. A. (2015). Removal of H2A.Z by INO80 promotes homologous recombination. EMBO Rep. 16, 986–994. 10.15252/embr.201540330 26142279 PMC4552491

[B4] Amir R. E. Van den Veyver I. B. Wan M. Tran C. Q. Francke U. Zoghbi H. Y. (1999). Rett syndrome is caused by mutations in X-linked MECP2, encoding methyl-CpG-binding protein 2. Nat. Genet. 23, 185–188. 10.1038/13810 10508514

[B5] Armache A. Yang S. Martínez de Paz A. Robbins L. E. Durmaz C. Cheong J. Q. (2020). Histone H3.3 phosphorylation amplifies stimulation-induced transcription. Nature 583, 852–857. 10.1038/s41586-020-2533-0 32699416 PMC7517595

[B6] Attar N. Kurdistani S. K. (2017). Exploitation of EP300 and CREBBP lysine acetyltransferases by cancer. Cold Spring Harb. Perspect. Med. 7, a026534. 10.1101/cshperspect.a026534 27881443 PMC5334244

[B7] Ausió J. Levin D. De Amorim G. Bakker S. Macleod P. (2003). Syndromes of disordered chromatin remodeling. Clin. Genet. 64, 83–95. 10.1034/j.1399-0004.2003.00124.x 12859401

[B8] Banaszynski L. A. Wen D. Dewell S. Whitcomb S. J. Lin M. Diaz N. (2013). Hira-dependent histone H3.3 deposition facilitates PRC2 recruitment at developmental loci in ES cells. Cell 155, 107–120. 10.1016/j.cell.2013.08.061 24074864 PMC3838450

[B9] Bargaje R. Alam M. P. Patowary A. Sarkar M. Ali T. Gupta S. (2012). Proximity of H2A.Z containing nucleosome to the transcription start site influences gene expression levels in the mammalian liver and brain. Nucleic Acids Res. 40, 8965–8978. 10.1093/nar/gks665 22821566 PMC3467062

[B10] Bayreuther K. Rodemann H. P. Hommel R. Dittmann K. Albiez M. Francz P. I. (1988). Human skin fibroblasts *in vitro* differentiate along a terminal cell lineage. Proc. Natl. Acad. Sci. U. S. A. 85, 5112–5116. 10.1073/pnas.85.14.5112 3393534 PMC281698

[B11] Begum N. A. Haque F. Stanlie A. Husain A. Mondal S. Nakata M. (2021). Phf5a regulates DNA repair in class switch recombination via p400 and histone H2A variant deposition. EMBO J. 40, e106393. 10.15252/embj.2020106393 33938017 PMC8204862

[B12] Bender S. Tang Y. Lindroth A. M. Hovestadt V. Jones D. T. W. Kool M. (2013). Reduced H3K27me3 and DNA hypomethylation are major drivers of gene expression in K27M mutant pediatric high-grade gliomas. Cancer Cell 24, 660–672. 10.1016/j.ccr.2013.10.006 24183680

[B13] Bodor D. L. Rodríguez M. G. Moreno N. Jansen L. E. T. (2012). Analysis of protein turnover by quantitative SNAP-based pulse-chase imaging. Curr. Protoc. Cell Biol. 55, 1–8. 10.1002/0471143030.cb0808s55 23129118

[B14] Boniel S. Szymańska K. Śmigiel R. Szczałuba K. (2021). Kabuki syndrome—clinical review with molecular aspects. Genes 12, 468. 10.3390/genes12040468 33805950 PMC8064399

[B15] Bosch A. Suau P. (1995). Changes in core histone variant composition in differentiating neurons: The roles of differential turnover and synthesis rates. Eur. J. Cell Biol. 68, 220–225. 8603674

[B16] Bourque G. Burns K. H. Gehring M. Gorbunova V. Seluanov A. Hammell M. (2018). Ten things you should know about transposable elements. Genome Biol. 19, 199. 10.1186/s13059-018-1577-z 30454069 PMC6240941

[B17] Buschbeck M. Hake S. B. (2017). Variants of core histones and their roles in cell fate decisions, development and cancer. Nat. Rev. Mol. Cell Biol. 18, 299–314. 10.1038/nrm.2016.166 28144029

[B18] Calcinotto A. Kohli J. Zagato E. Pellegrini L. Demaria M. Alimonti A. (2019). Cellular senescence: Aging, cancer, and injury. Physiol. Rev. 99, 1047–1078. 10.1152/physrev.00020.2018 30648461

[B19] Caron P. Pobega E. Polo S. E. (2021). DNA double-strand break repair: All roads lead to HeterochROMAtin marks. Front. Genet. 12. 730696, 10.3389/fgene.2021.730696 34539757 PMC8440905

[B20] Chakraborty U. Shen Z.-J. Tyler J. (2021). Chaperoning histones at the DNA repair dance. DNA Repair 108, 103240. 10.1016/j.dnarep.2021.103240 34687987 PMC8827131

[B21] Chaudhury I. Sareen A. Raghunandan M. Sobeck A. (2013). FANCD2 regulates BLM complex functions independently of FANCI to promote replication fork recovery. Nucleic Acids Res. 41, 6444–6459. 10.1093/nar/gkt348 23658231 PMC3711430

[B22] Chen C. Sun M. Warzecha C. Bachu M. Dey A. Wu T. (2020a). HIRA, a DiGeorge syndrome candidate gene, confers proper chromatin accessibility on HSCs and supports all stages of hematopoiesis. Cell Rep. 30, 2136–2149. e4. 10.1016/j.celrep.2020.01.062 32075733

[B23] Chen F. Zhang W. Xie D. Gao T. Dong Z. Lu X. (2020b). Histone chaperone FACT represses retrotransposon MERVL and MERVL-derived cryptic promoters. Nucleic Acids Res. 48, 10211–10225. 10.1093/nar/gkaa732 32894293 PMC7544220

[B24] Chen P. Dong L. Hu M. Wang Y.-Z. Xiao X. Zhao Z. (2018). Functions of FACT in breaking the nucleosome and maintaining its integrity at the single-nucleosome level. Mol. Cell 71, 284–293. e4. 10.1016/j.molcel.2018.06.020 30029006

[B25] Chen P. Zhao J. Wang Y. Wang M. Long H. Liang D. (2013). H3.3 actively marks enhancers and primes gene transcription via opening higher-ordered chromatin. Genes Dev. 27, 2109–2124. 10.1101/gad.222174.113 24065740 PMC3850095

[B26] Chen Y. Chen Q. McEachin R. C. Cavalcoli J. D. Yu X. (2014). H2A.B facilitates transcription elongation at methylated CpG loci. Genome Res. 24, 570–579. 10.1101/gr.156877.113 24402521 PMC3975057

[B27] Clément C. Orsi G. A. Gatto A. Boyarchuk E. Forest A. Hajj B. (2018). High-resolution visualization of H3 variants during replication reveals their controlled recycling. Nat. Commun. 9, 3181. 10.1038/s41467-018-05697-1 30093638 PMC6085313

[B28] Clément C. Vassias I. Ray-Gallet D. Almouzni G. (2016). Functional characterization of histone chaperones using SNAP-tag-based imaging to assess de novo histone deposition. Methods Enzymol. 573, 97–117. 10.1016/bs.mie.2016.04.004 27372750

[B29] Contrepois K. Coudereau C. Benayoun B. A. Schuler N. Roux P.-F. Bischof O. (2017). Histone variant H2A.J accumulates in senescent cells and promotes inflammatory gene expression. Nat. Commun. 8, 14995. 10.1038/ncomms14995 28489069 PMC5436145

[B30] Coppé J.-P. Patil C. K. Rodier F. Sun Y. Muñoz D. P. Goldstein J. (2008). Senescence-associated secretory phenotypes reveal cell-nonautonomous functions of oncogenic RAS and the p53 tumor suppressor. PLoS Biol. 6, 2853–2868. 10.1371/journal.pbio.0060301 19053174 PMC2592359

[B31] Costanzi C. Pehrson J. R. (1998). Histone macroH2A1 is concentrated in the inactive X chromosome of female mammals. Nature 393, 599–601. 10.1038/31275 9634239

[B32] Dahl J. A. Gilfillan G. D. (2018). How low can you go? Pushing the limits of low-input ChIP-seq. Brief. Funct. Genomics 17, 89–95. 10.1093/bfgp/elx037 29087438

[B33] D’Antoni S. Mattina T. Di Mare P. Federico C. Motta S. Saccone S. (2004). Altered replication timing of the HIRA/Tuple1 locus in the DiGeorge and Velocardiofacial syndromes. Gene 333, 111–119. 10.1016/j.gene.2004.02.029 15177686

[B34] Dash R. C. Zaino A. M. Hadden M. K. (2018). A metadynamic approach to understand the recognition mechanism of the histone H3 tail with the ATRXADD domain. Biochim. Biophys. Acta. Gene Regul. Mech. 1861, 594–602. 10.1016/j.bbagrm.2018.05.001 29730439

[B35] De Koning L. Corpet A. Haber J. E. Almouzni G. (2007). Histone chaperones: An escort network regulating histone traffic. Nat. Struct. Mol. Biol. 14, 997–1007. 10.1038/nsmb1318 17984962

[B36] Deininger P. L. Moran J. V. Batzer M. A. Kazazian H. H. (2003). Mobile elements and mammalian genome evolution. Curr. Opin. Genet. Dev. 13, 651–658. 10.1016/j.gde.2003.10.013 14638329

[B37] Delaunoy J. Abidi F. Zeniou M. Jacquot S. Merienne K. Pannetier S. (2001). Mutations in the X-linked RSK2 gene (RPS6KA3) in patients with Coffin-Lowry syndrome. Hum. Mutat. 17, 103–116. 10.1002/1098-1004(200102)17:2<103::AID-HUMU2>3.0.CO;2-N 11180593

[B38] Dimri G. P. Lee X. Basile G. Acosta M. Scott G. Roskelley C. (1995). A biomarker that identifies senescent human cells in culture and in aging skin *in vivo* . Proc. Natl. Acad. Sci. U. S. A. 92, 9363–9367. 10.1073/pnas.92.20.9363 7568133 PMC40985

[B39] Dodsworth S. Chase M. W. Kelly L. J. Leitch I. J. Macas J. Novák P. (2015). Genomic repeat abundances contain phylogenetic signal. Syst. Biol. 64, 112–126. 10.1093/sysbio/syu080 25261464 PMC4265144

[B40] Drané P. Ouararhni K. Depaux A. Shuaib M. Hamiche A. (2010). The death-associated protein DAXX is a novel histone chaperone involved in the replication-independent deposition of H3.3. Genes Dev. 24, 1253–1265. 10.1101/gad.566910 20504901 PMC2885661

[B41] Duarte L. F. Young A. R. J. Wang Z. Wu H.-A. Panda T. Kou Y. (2014). Histone H3.3 and its proteolytically processed form drive a cellular senescence programme. Nat. Commun. 5, 5210. 10.1038/ncomms6210 25394905 PMC4235654

[B42] Dunn C. J. Sarkar P. Bailey E. R. Farris S. Zhao M. Ward J. M. (2017). Histone hypervariants H2A.Z.1 and H2A.Z.2 play independent and context-specific roles in neuronal activity-induced transcription of arc/arg3.1 and other immediate early genes. eNeuro 4, ENEURO.0040–17.2017. 10.1523/ENEURO.0040-17.2017 28856239 PMC5569379

[B43] Dyer M. A. Qadeer Z. A. Valle-Garcia D. Bernstein E. (2017). ATRX and DAXX: Mechanisms and mutations. Cold Spring Harb. Perspect. Med. 7, a026567. 10.1101/cshperspect.a026567 28062559 PMC5334245

[B44] Elsaesser S. J. Allis C. D. (2010). HIRA and Daxx constitute two independent histone H3.3-containing predeposition complexes. Cold Spring Harb. Symp. Quant. Biol. 75, 27–34. 10.1101/sqb.2010.75.008 21047901

[B46] Elsässer S. J. Noh K.-M. Diaz N. Allis C. D. Banaszynski L. A. (2015). Histone H3.3 is required for endogenous retroviral element silencing in embryonic stem cells. Nature 522, 240–244. 10.1038/nature14345 25938714 PMC4509593

[B47] Esteves de Lima J. Bou Akar R. Machado L. Li Y. Drayton-Libotte B. Dilworth F. J. (2021). HIRA stabilizes skeletal muscle lineage identity. Nat. Commun. 12, 3450. 10.1038/s41467-021-23775-9 34103504 PMC8187366

[B48] Farrell M. J. Stadt H. Wallis K. T. Scambler P. Hixon R. L. Wolfe R. (1999). HIRA, a DiGeorge syndrome candidate gene, is required for cardiac outflow tract septation. Circ. Res. 84, 127–135. 10.1161/01.RES.84.2.127 9933243

[B49] Felsenfeld G. Groudine M. (2003). Controlling the double helix. Nature 421, 448–453. 10.1038/nature01411 12540921

[B50] Ferrand J. Rondinelli B. Polo S. E. (2020). Histone variants: Guardians of genome integrity. Cells 9, 2424. 10.3390/cells9112424 33167489 PMC7694513

[B51] Feschotte C. Gilbert C. (2012). Endogenous viruses: Insights into viral evolution and impact on host biology. Nat. Rev. Genet. 13, 283–296. 10.1038/nrg3199 22421730

[B52] Finnegan D. J. (1989). Eukaryotic transposable elements and genome evolution. Trends Genet. 5, 103–107. 10.1016/0168-9525(89)90039-5 2543105

[B53] Fortuny A. Chansard A. Caron P. Chevallier O. Leroy O. Renaud O. (2021). Imaging the response to DNA damage in heterochromatin domains reveals core principles of heterochromatin maintenance. Nat. Commun. 12, 2428. 10.1038/s41467-021-22575-5 33893291 PMC8065061

[B54] Frey A. Listovsky T. Guilbaud G. Sarkies P. Sale J. E. (2014). Histone H3.3 is required to maintain replication fork progression after UV damage. Curr. Biol. 24, 2195–2201. 10.1016/j.cub.2014.07.077 25201682 PMC4175177

[B55] Fülöp N. Feng W. Xing D. He K. Nőt L. G. Brocks C. A. (2008). Aging leads to increased levels of protein O-linked N-acetylglucosamine in heart, aorta, brain and skeletal muscle in Brown-Norway rats. Biogerontology 9, 139–151. 10.1007/s10522-007-9123-5 18185980 PMC2810282

[B56] Fyodorov D. V. Zhou B.-R. Skoultchi A. I. Bai Y. (2018). Emerging roles of linker histones in regulating chromatin structure and function. Nat. Rev. Mol. Cell Biol. 19, 192–206. 10.1038/nrm.2017.94 29018282 PMC5897046

[B57] Gamble M. J. Frizzell K. M. Yang C. Krishnakumar R. Kraus W. L. (2010). The histone variant macroH2A1 marks repressed autosomal chromatin, but protects a subset of its target genes from silencing. Genes Dev. 24, 21–32. 10.1101/gad.1876110 20008927 PMC2802188

[B58] Gatto A. Forest A. Quivy J.-P. Almouzni G. (2022). HIRA-dependent boundaries between H3 variants shape early replication in mammals. Mol. Cell 82, 1909–1923.e5. e5. 10.1016/j.molcel.2022.03.017 35381196

[B59] Ghiraldini F. G. Filipescu D. Bernstein E. (2021). Solid tumours hijack the histone variant network. Nat. Rev. Cancer 21, 257–275. 10.1038/s41568-020-00330-0 33568791 PMC8092022

[B60] Goldberg A. D. Banaszynski L. A. Noh K.-M. Lewis P. W. Elsaesser S. J. Stadler S. (2010). Distinct factors control histone variant H3.3 localization at specific genomic regions. Cell 140, 678–691. 10.1016/j.cell.2010.01.003 20211137 PMC2885838

[B61] Groh S. Milton A. V. Marinelli L. K. Sickinger C. V. Russo A. Bollig H. (2021). Morc3 silences endogenous retroviruses by enabling Daxx-mediated histone H3.3 incorporation. Nat. Commun. 12, 5996. 10.1038/s41467-021-26288-7 34650047 PMC8516933

[B62] Grover P. Asa J. S. Campos E. I. (2018). H3–H4 histone chaperone pathways. Annu. Rev. Genet. 52, 109–130. 10.1146/annurev-genet-120417-031547 30183406

[B63] Gulve N. Su C. Deng Z. Soldan S. S. Vladimirova O. Wickramasinghe J. (2022). DAXX-ATRX regulation of p53 chromatin binding and DNA damage response. Nat. Commun. 13, 5033. 10.1038/s41467-022-32680-8 36028493 PMC9418176

[B64] Guo R. Zheng L. Park J. W. Lv R. Chen H. Jiao F. (2014). BS69/ZMYND11 reads and connects histone H3.3 lysine 36 trimethylation-decorated chromatin to regulated pre-mRNA processing. Mol. Cell 56, 298–310. 10.1016/j.molcel.2014.08.022 25263594 PMC4363072

[B65] Gurard-Levin Z. A. Quivy J.-P. Almouzni G. (2014). Histone chaperones: Assisting histone traffic and nucleosome dynamics. Annu. Rev. Biochem. 83, 487–517. 10.1146/annurev-biochem-060713-035536 24905786

[B66] Gursoy-Yuzugullu O. Ayrapetov M. K. Price B. D. (2015). Histone chaperone Anp32e removes H2A.Z from DNA double-strand breaks and promotes nucleosome reorganization and DNA repair. Proc. Natl. Acad. Sci. U. S. A. 112, 7507–7512. 10.1073/pnas.1504868112 26034280 PMC4475971

[B67] Hake S. B. Garcia B. A. Kauer M. Baker S. P. Shabanowitz J. Hunt D. F. (2005). Serine 31 phosphorylation of histone variant H3.3 is specific to regions bordering centromeres in metaphase chromosomes. Proc. Natl. Acad. Sci. U. S. A. 102, 6344–6349. 10.1073/pnas.0502413102 15851689 PMC1088391

[B68] Hammond C. M. Strømme C. B. Huang H. Patel D. J. Groth A. (2017). Histone chaperone networks shaping chromatin function. Nat. Rev. Mol. Cell Biol. 18, 141–158. 10.1038/nrm.2016.159 28053344 PMC5319910

[B69] Hardy S. Jacques P.-É. Gévry N. Forest A. Fortin M.-È. Laflamme L. (2009). The euchromatic and heterochromatic landscapes are shaped by antagonizing effects of transcription on H2A.Z deposition. PLoS Genet. 5, e1000687. 10.1371/journal.pgen.1000687 19834540 PMC2754525

[B70] Hatanaka Y. Inoue K. Oikawa M. Kamimura S. Ogonuki N. Kodama E. N. (2015). Histone chaperone CAF-1 mediates repressive histone modifications to protect preimplantation mouse embryos from endogenous retrotransposons. Proc. Natl. Acad. Sci. U. S. A. 112, 14641–14646. 10.1073/pnas.1512775112 26546670 PMC4664303

[B71] He Q. Kim H. Huang R. Lu W. Tang M. Shi F. (2015). The daxx/atrx complex protects Tandem repetitive elements during DNA hypomethylation by promoting H3K9 trimethylation. Cell Stem Cell 17, 273–286. 10.1016/j.stem.2015.07.022 26340527 PMC4571182

[B72] Hédouin S. Grillo G. Ivkovic I. Velasco G. Francastel C. (2017). CENP-A chromatin disassembly in stressed and senescent murine cells. Sci. Rep. 7, 42520. 10.1038/srep42520 28186195 PMC5301216

[B73] Hellsten U. Harland R. M. Gilchrist M. J. Hendrix D. Jurka J. Kapitonov V. (2010). The genome of the western clawed frog Xenopus tropicalis. Science 328, 633–636. 10.1126/science.1183670 20431018 PMC2994648

[B74] Hernandez-Segura A. Nehme J. Demaria M. (2018). Hallmarks of cellular senescence. Trends Cell Biol. 28, 436–453. 10.1016/j.tcb.2018.02.001 29477613

[B75] Herranz N. Gil J. (2018). Mechanisms and functions of cellular senescence. J. Clin. Invest. 128, 1238–1246. 10.1172/JCI95148 29608137 PMC5873888

[B76] Hoelper D. Huang H. Jain A. Y. Patel D. J. Lewis P. W. (2017). Structural and mechanistic insights into ATRX-dependent and -independent functions of the histone chaperone DAXX. Nat. Commun. 8, 1193. 10.1038/s41467-017-01206-y 29084956 PMC5662737

[B77] Huang H. Strømme C. B. Saredi G. Hödl M. Strandsby A. González-Aguilera C. (2015). A unique binding mode enables MCM2 to chaperone histones H3–H4 at replication forks. Nat. Struct. Mol. Biol. 22, 618–626. 10.1038/nsmb.3055 26167883 PMC4685956

[B78] Ishiuchi T. Enriquez-Gasca R. Mizutani E. Bošković A. Ziegler-Birling C. Rodriguez-Terrones D. (2015). Early embryonic-like cells are induced by downregulating replication-dependent chromatin assembly. Nat. Struct. Mol. Biol. 22, 662–671. 10.1038/nsmb.3066 26237512

[B79] Isogai Y. Keles S. Prestel M. Hochheimer A. Tjian R. (2007). Transcription of histone gene cluster by differential core-promoter factors. Genes Dev. 21, 2936–2949. 10.1101/gad.1608807 17978101 PMC2049195

[B80] Jangam D. Feschotte C. Betrán E. (2017). Transposable element domestication as an adaptation to evolutionary conflicts. Trends Genet. 33, 817–831. 10.1016/j.tig.2017.07.011 28844698 PMC5659911

[B81] Jeanne M. Vuillaume M.-L. Ung D. C. Vancollie V. E. Wagner C. Collins S. C. (2021). Haploinsufficiency of the HIRA gene located in the 22q11 deletion syndrome region is associated with abnormal neurodevelopment and impaired dendritic outgrowth. Hum. Genet. 140, 885–896. 10.1007/s00439-020-02252-1 33417013

[B82] Jeffery D. Podsypanina K. Yadav T. Almouzni G. (2019). “Chromatin dynamics in cancer: Epigenetic parameters and cellular fate,” in Encyclopedia of cancer. Editors Boffetta P. Hainaut P.. Third Edition (Oxford: Academic Press), 372–388. 10.1016/B978-0-12-801238-3.65276-5)

[B83] Jimeno-González S. Payán-Bravo L. Muñoz-Cabello A. M. Guijo M. Gutierrez G. Prado F. (2015). Defective histone supply causes changes in RNA polymerase II elongation rate and cotranscriptional pre-mRNA splicing. Proc. Natl. Acad. Sci. U. S. A. 112, 14840–14845. 10.1073/pnas.1506760112 26578803 PMC4672771

[B84] Jin C. Zang C. Wei G. Cui K. Peng W. Zhao K. (2009). H3.3/H2A.Z double variant–containing nucleosomes mark “nucleosome-free regions” of active promoters and other regulatory regions. Nat. Genet. 41, 941–945. 10.1038/ng.409 19633671 PMC3125718

[B85] Karimi M. M. Goyal P. Maksakova I. A. Bilenky M. Leung D. Tang J. X. (2011). DNA methylation and SETDB1/H3K9me3 regulate predominantly distinct sets of genes, retroelements, and chimeric transcripts in mESCs. Cell Stem Cell 8, 676–687. 10.1016/j.stem.2011.04.004 21624812 PMC3857791

[B86] Keppler A. Gendreizig S. Gronemeyer T. Pick H. Vogel H. Johnsson K. (2003). A general method for the covalent labeling of fusion proteins with small molecules *in vivo* . Nat. Biotechnol. 21, 86–89. 10.1038/nbt765 12469133

[B87] Kuo M. H. Allis C. D. (1999). *In vivo* cross-linking and immunoprecipitation for studying dynamic Protein:DNA associations in a chromatin environment. Methods 19, 425–433. 10.1006/meth.1999.0879 10579938

[B88] Latreille D. Bluy L. Benkirane M. Kiernan R. E. (2014). Identification of histone 3 variant 2 interacting factors. Nucleic Acids Res. 42, 3542–3550. 10.1093/nar/gkt1355 24393775 PMC3973350

[B89] Lee J.-S. Zhang Z. (2016). O-linked N-acetylglucosamine transferase (OGT) interacts with the histone chaperone HIRA complex and regulates nucleosome assembly and cellular senescence. Proc. Natl. Acad. Sci. U. S. A. 113, E3213–E3220. 10.1073/pnas.1600509113 27217568 PMC4988580

[B90] Lee J. Han K. Meyer T. J. Kim H.-S. Batzer M. A. (2008). Chromosomal inversions between human and chimpanzee lineages caused by retrotransposons. PLOS ONE 3, e4047. 10.1371/journal.pone.0004047 19112500 PMC2603318

[B91] Lepack A. E. Bagot R. C. Peña C. J. Loh Y.-H. E. Farrelly L. A. Lu Y. (2016). Aberrant H3.3 dynamics in NAc promote vulnerability to depressive-like behavior. Proc. Natl. Acad. Sci. U. S. A. 113, 12562–12567. 10.1073/pnas.1608270113 27791098 PMC5098673

[B92] Leplae R. Hebrant A. Wodak S. J. Toussaint A. (2004). Aclame: A CLAssification of mobile genetic elements. Nucleic Acids Res. 32, D45–D49. 10.1093/nar/gkh084 14681355 PMC308818

[B93] Lewis P. W. Elsaesser S. J. Noh K.-M. Stadler S. C. Allis C. D. (2010). Daxx is an H3.3-specific histone chaperone and cooperates with ATRX in replication-independent chromatin assembly at telomeres. Proc. Natl. Acad. Sci. U. S. A. 107, 14075–14080. 10.1073/pnas.1008850107 20651253 PMC2922592

[B95] Liu W. H. Roemer S. C. Zhou Y. Shen Z.-J. Dennehey B. K. Balsbaugh J. L. (2016). The Cac1 subunit of histone chaperone CAF-1 organizes CAF-1-H3/H4 architecture and tetramerizes histones. eLife 5, e18023. 10.7554/eLife.18023 27690308 PMC5045291

[B96] Liu Y. Zhou K. Zhang N. Wei H. Tan Y. Z. Zhang Z. (2020). FACT caught in the act of manipulating the nucleosome. Nature 577, 426–431. 10.1038/s41586-019-1820-0 31775157 PMC7441595

[B97] Long H. Zhang L. Lv M. Wen Z. Zhang W. Chen X. (2020). H2A.Z facilitates licensing and activation of early replication origins. Nature 577, 576–581. 10.1038/s41586-019-1877-9 31875854

[B98] Longo M. S. Carone D. M. Green E. D. O’Neill M. J. O’Neill R. J. NISC Comparative Sequencing Program (2009). Distinct retroelement classes define evolutionary breakpoints demarcating sites of evolutionary novelty. BMC Genomics 10, 334. 10.1186/1471-2164-10-334 19630942 PMC2736999

[B99] Luger K. Mäder A. W. Richmond R. K. Sargent D. F. Richmond T. J. (1997). Crystal structure of the nucleosome core particle at 2.8 Å resolution. Nature 389, 251–260. 10.1038/38444 9305837

[B100] Macfarlan T. S. Gifford W. D. Driscoll S. Lettieri K. Rowe H. M. Bonanomi D. (2012). Embryonic stem cell potency fluctuates with endogenous retrovirus activity. Nature 487, 57–63. 10.1038/nature11244 22722858 PMC3395470

[B101] Maciel-Barón L. Á. Moreno-Blas D. Morales-Rosales S. L. González-Puertos V. Y. López-Díazguerrero N. E. Torres C. (2018). Cellular senescence, neurological function, and redox state. Antioxid. Redox Signal. 28, 1704–1723. 10.1089/ars.2017.7112 28467755

[B102] Maksakova I. A. Mager D. L. Reiss D. (2008). Keeping active endogenous retroviral-like elements in check: The epigenetic perspective. Cell. Mol. Life Sci. 65, 3329–3347. 10.1007/s00018-008-8494-3 18818875 PMC11131743

[B103] Mao Z. Pan L. Wang W. Sun J. Shan S. Dong Q. (2014). Anp32e, a higher eukaryotic histone chaperone directs preferential recognition for H2A.Z. Cell Res. 24, 389–399. 10.1038/cr.2014.30 24613878 PMC3975505

[B104] Martire S. Banaszynski L. A. (2020). The roles of histone variants in fine-tuning chromatin organization and function. Nat. Rev. Mol. Cell Biol. 21, 522–541. 10.1038/s41580-020-0262-8 32665685 PMC8245300

[B105] Martire S. Gogate A. A. Whitmill A. Tafessu A. Nguyen J. Teng Y.-C. (2019). Phosphorylation of histone H3.3 at serine 31 promotes p300 activity and enhancer acetylation. Nat. Genet. 51, 941–946. 10.1038/s41588-019-0428-5 31152160 PMC6598431

[B106] Marzluff W. F. Koreski K. P. (2017). Birth and death of histone mRNAs. Trends Genet. 33, 745–759. 10.1016/j.tig.2017.07.014 28867047 PMC5645032

[B107] Marzluff W. F. Wagner E. J. Duronio R. J. (2008). Metabolism and regulation of canonical histone mRNAs: Life without a poly(A) tail. Nat. Rev. Genet. 9, 843–854. 10.1038/nrg2438 18927579 PMC2715827

[B108] Matsui T. Leung D. Miyashita H. Maksakova I. A. Miyachi H. Kimura H. (2010). Proviral silencing in embryonic stem cells requires the histone methyltransferase ESET. Nature 464, 927–931. 10.1038/nature08858 20164836

[B109] Maze I. Wenderski W. Noh K.-M. Bagot R. C. Tzavaras N. Purushothaman I. (2015). Critical role of histone turnover in neuronal transcription and plasticity. Neuron 87, 77–94. 10.1016/j.neuron.2015.06.014 26139371 PMC4491146

[B110] McDonald-McGinn D. M. Sullivan K. E. Marino B. Philip N. Swillen A. Vorstman J. A. S. (2015). 22q11.2 deletion syndrome. Nat. Rev. Dis. Prim. 1, 15071–15119. 10.1038/nrdp.2015.71 27189754 PMC4900471

[B111] McNally A. G. Poplawski S. G. Mayweather B. A. White K. M. Abel T. (2016). Characterization of a novel chromatin sorting tool reveals importance of histone variant H3.3 in contextual fear memory and motor learning. Front. Mol. Neurosci. 9. 11, 10.3389/fnmol.2016.00011 26903803 PMC4746260

[B112] Mei Q. Huang J. Chen W. Tang J. Xu C. Yu Q. (2017). Regulation of DNA replication-coupled histone gene expression. Oncotarget 8, 95005–95022. 10.18632/oncotarget.21887 29212286 PMC5706932

[B113] Michod D. Bartesaghi S. Khelifi A. Bellodi C. Berliocchi L. Nicotera P. (2012). Calcium-dependent dephosphorylation of the histone chaperone DAXX regulates H3.3 loading and transcription upon neuronal activation. Neuron 74, 122–135. 10.1016/j.neuron.2012.02.021 22500635 PMC3657165

[B114] Moreno-Blas D. Gorostieta-Salas E. Castro-Obregón S. (2018). Connecting chaperone-mediated autophagy dysfunction to cellular senescence. Ageing Res. Rev. 41, 34–41. 10.1016/j.arr.2017.11.001 29113832

[B115] Mossink B. Negwer M. Schubert D. Nadif Kasri N. (2021). The emerging role of chromatin remodelers in neurodevelopmental disorders: A developmental perspective. Cell. Mol. Life Sci. 78, 2517–2563. 10.1007/s00018-020-03714-5 33263776 PMC8004494

[B116] Murata T. Kurokawa R. Krones A. Tatsumi K. Ishii M. Taki T. (2001). Defect of histone acetyltransferase activity of the nuclear transcriptional coactivator CBP in Rubinstein–Taybi syndrome. Hum. Mol. Genet. 10, 1071–1076. 10.1093/hmg/10.10.1071 11331617

[B117] Murray K. (1966). The acid extraction of histones from calf thymus deoxyribonucleoprotein. J. Mol. Biol. 15, 409–419. 10.1016/S0022-2836(66)80116-X 5915175

[B118] Nacev B. A. Feng L. Bagert J. D. Lemiesz A. E. Gao J. Soshnev A. A. (2019). The expanding landscape of “oncohistone” mutations in human cancers. Nature 567, 473–478. 10.1038/s41586-019-1038-1 30894748 PMC6512987

[B119] Nepal M. Che R. Zhang J. Ma C. Fei P. (2017). Fanconi anemia signaling and cancer. Trends Cancer 3, 840–856. 10.1016/j.trecan.2017.10.005 29198440 PMC5819365

[B120] Nye J. Melters D. P. Dalal Y. (2018). The art of war: Harnessing the epigenome against cancer. F1000Res. 7, 141. 10.12688/f1000research.12833.1 29479426 PMC5801563

[B121] Obri A. Ouararhni K. Papin C. Diebold M.-L. Padmanabhan K. Marek M. (2014). ANP32E is a histone chaperone that removes H2A.Z from chromatin. Nature 505, 648–653. 10.1038/nature12922 24463511

[B122] Orphanides G. Wu W.-H. Lane W. S. Hampsey M. Reinberg D. (1999). The chromatin-specific transcription elongation factor FACT comprises human SPT16 and SSRP1 proteins. Nature 400, 284–288. 10.1038/22350 10421373

[B123] Papamichos-Chronakis M. Watanabe S. Rando O. J. Peterson C. L. (2011). Global regulation of H2A.Z localization by the INO80 chromatin-remodeling enzyme is essential for genome integrity. Cell 144, 200–213. 10.1016/j.cell.2010.12.021 21241891 PMC3035940

[B124] Petryk N. Reverón-Gómez N. González-Aguilera C. Dalby M. Andersson R. Groth A. (2021). Genome-wide and sister chromatid-resolved profiling of protein occupancy in replicated chromatin with ChOR-seq and SCAR-seq. Nat. Protoc. 16, 4446–4493. 10.1038/s41596-021-00585-3 34363071

[B125] Piña B. Suau P. (1987). Changes in histones H2A and H3 variant composition in differentiating and mature rat brain cortical neurons. Dev. Biol. 123, 51–58. 10.1016/0012-1606(87)90426-X 3622934

[B126] Piquet S. Parc F. L. Bai S.-K. Chevallier O. Adam S. Polo S. E. (2018). The histone chaperone FACT coordinates H2A.X-dependent signaling and repair of DNA damage. Mol. Cell 72, 888–901. e7. 10.1016/j.molcel.2018.09.010 30344095 PMC6292839

[B127] Rafiee M.-R. Girardot C. Sigismondo G. Krijgsveld J. (2016). Expanding the circuitry of pluripotency by selective isolation of chromatin-associated proteins. Mol. Cell 64, 624–635. 10.1016/j.molcel.2016.09.019 27773674 PMC5101186

[B128] Rai T. S. Adams P. D. (2012). Lessons from senescence: Chromatin maintenance in non-proliferating cells. Biochim. Biophys. Acta 1819, 322–331. 10.1016/j.bbagrm.2011.07.014 21839870 PMC3895594

[B129] Ramzan F. Creighton S. D. Hall M. Baumbach J. Wahdan M. Poulson S. J. (2020). Sex-specific effects of the histone variant H2A.Z on fear memory, stress-enhanced fear learning and hypersensitivity to pain. Sci. Rep. 10, 14331. 10.1038/s41598-020-71229-x 32868857 PMC7458907

[B130] Ray-Gallet D. Quivy J.-P. Scamps C. Martini E. M.-D. Lipinski M. Almouzni G. (2002). HIRA is critical for a nucleosome assembly pathway independent of DNA synthesis. Mol. Cell 9, 1091–1100. 10.1016/s1097-2765(02)00526-9 12049744

[B131] Ricci M. Peona V. Guichard E. Taccioli C. Boattini A. (2018). Transposable elements activity is positively related to rate of speciation in mammals. J. Mol. Evol. 86, 303–310. 10.1007/s00239-018-9847-7 29855654 PMC6028844

[B132] Robberecht C. Voet T. Esteki M. Z. Nowakowska B. A. Vermeesch J. R. (2013). Nonallelic homologous recombination between retrotransposable elements is a driver of de novo unbalanced translocations. Genome Res. 23, 411–418. 10.1101/gr.145631.112 23212949 PMC3589530

[B133] Roberts C. Daw S. C. M. Halford S. Scambler P. J. (1997). Cloning and developmental expression analysis of chick hira (chira), a candidate gene for DiGeorge syndrome. Hum. Mol. Genet. 6, 237–245. 10.1093/hmg/6.2.237 9063744

[B134] Rodier F. Muñoz D. P. Teachenor R. Chu V. Le O. Bhaumik D. (2011). DNA-SCARS: Distinct nuclear structures that sustain damage-induced senescence growth arrest and inflammatory cytokine secretion. J. Cell Sci. 124, 68–81. 10.1242/jcs.071340 21118958 PMC3001408

[B135] Rodriguez F. Arkhipova I. R. (2018). Transposable elements and polyploid evolution in animals. Curr. Opin. Genet. Dev. 49, 115–123. 10.1016/j.gde.2018.04.003 29715568 PMC5975190

[B136] Rogakou E. P. Pilch D. R. Orr A. H. Ivanova V. S. Bonner W. M. (1998). DNA double-stranded breaks induce histone H2AX phosphorylation on serine 139. J. Biol. Chem. 273, 5858–5868. 10.1074/jbc.273.10.5858 9488723

[B137] Rowe H. M. Jakobsson J. Mesnard D. Rougemont J. Reynard S. Aktas T. (2010). KAP1 controls endogenous retroviruses in embryonic stem cells. Nature 463, 237–240. 10.1038/nature08674 20075919

[B138] Rowe H. M. Kapopoulou A. Corsinotti A. Fasching L. Macfarlan T. S. Tarabay Y. (2013). TRIM28 repression of retrotransposon-based enhancers is necessary to preserve transcriptional dynamics in embryonic stem cells. Genome Res. 23, 452–461. 10.1101/gr.147678.112 23233547 PMC3589534

[B139] Saade E. Pirozhkova I. Aimbetov R. Lipinski M. Ogryzko V. (2015). Molecular turnover, the H3.3 dilemma and organismal aging (hypothesis). Aging Cell 14, 322–333. 10.1111/acel.12332 25720734 PMC4406661

[B140] Sadic D. Schmidt K. Groh S. Kondofersky I. Ellwart J. Fuchs C. (2015). Atrx promotes heterochromatin formation at retrotransposons. EMBO Rep. 16, 836–850. 10.15252/embr.201439937 26012739 PMC4515123

[B141] Sansoni V. Casas-Delucchi C. S. Rajan M. Schmidt A. Bönisch C. Thomae A. W. (2014). The histone variant H2A.Bbd is enriched at sites of DNA synthesis. Nucleic Acids Res. 42, 6405–6420. 10.1093/nar/gku303 24753410 PMC4041467

[B142] Schwartz B. E. Ahmad K. (2005). Transcriptional activation triggers deposition and removal of the histone variant H3.3. Genes Dev. 19, 804–814. 10.1101/gad.1259805 15774717 PMC1074318

[B143] Scott W. A. Campos E. I. (2020). Interactions with histone H3 & tools to study them. Front. Cell Dev. Biol. 8, 701. 10.3389/fcell.2020.00701 32850821 PMC7411163

[B144] Shechter D. Dormann H. L. Allis C. D. Hake S. B. (2007). Extraction, purification and analysis of histones. Nat. Protoc. 2, 1445–1457. 10.1038/nprot.2007.202 17545981

[B145] Siefert J. L. (2009). “Defining the mobilome,” in Horizontal gene transfer: *Genomes in flux* methods in molecular biology. Editors Gogarten M. B. Gogarten J. P. Olendzenski L. C. (Totowa, NJ: Humana Press), 13–27. 10.1007/978-1-60327-853-9_2

[B146] Sivkina A. L. Karlova M. G. Valieva M. E. McCullough L. L. Formosa T. Shaytan A. K. (2022). Electron microscopy analysis of ATP-independent nucleosome unfolding by FACT. Commun. Biol. 5, 2–9. 10.1038/s42003-021-02948-8 35013515 PMC8748794

[B147] Soboleva T. A. Parker B. J. Nekrasov M. Hart-Smith G. Tay Y. J. Tng W.-Q. (2017). A new link between transcriptional initiation and pre-mRNA splicing: The RNA binding histone variant H2A.B. PLOS Genet. 13, e1006633. 10.1371/journal.pgen.1006633 28234895 PMC5345878

[B148] Stefanelli G. Azam A. B. Walters B. J. Brimble M. A. Gettens C. P. Bouchard-Cannon P. (2018). Learning and age-related changes in genome-wide H2A.Z binding in the mouse Hippocampus. Cell Rep. 22, 1124–1131. 10.1016/j.celrep.2018.01.020 29386101 PMC5820781

[B149] Sullivan K. D. Mullen T. E. Marzluff W. F. Wagner E. J. (2009). Knockdown of SLBP results in nuclear retention of histone mRNA. RNA N. Y. N. 15, 459–472. 10.1261/rna.1205409 PMC2657014 19155325

[B150] Sultana S. Zarreen F. Chakraborty S. (2021). Insights into the roles of histone chaperones in nucleosome assembly and disassembly in virus infection. Virus Res. 297, 198395. 10.1016/j.virusres.2021.198395 33737155

[B151] Sutcliffe E. L. Parish I. A. He Y. Q. Juelich T. Tierney M. L. Rangasamy D. (2009). Dynamic histone variant exchange accompanies gene induction in T cells. Mol. Cell. Biol. 29, 1972–1986. 10.1128/MCB.01590-08 19158270 PMC2655607

[B152] Tagami H. Ray-Gallet D. Almouzni G. Nakatani Y. (2004). Histone H3.1 and H3.3 complexes mediate nucleosome assembly pathways dependent or independent of DNA synthesis. Cell 116, 51–61. 10.1016/s0092-8674(03)01064-x 14718166

[B153] Tolstorukov M. Y. Goldman J. A. Gilbert C. Ogryzko V. Kingston R. E. Park P. J. (2012). Histone variant H2A.Bbd is associated with active transcription and mRNA processing in human cells. Mol. Cell 47, 596–607. 10.1016/j.molcel.2012.06.011 22795134 PMC3708478

[B154] Torné J. Orsi G. A. Ray-Gallet D. Almouzni G. (2018). Imaging newly synthesized and old histone variant dynamics dependent on chaperones using the SNAP-tag system. Methods Mol. Biol. 1832, 207–221. 10.1007/978-1-4939-8663-7_11 30073529

[B155] Torné J. Ray-Gallet D. Boyarchuk E. Garnier M. Le Baccon P. Coulon A. (2020). Two HIRA-dependent pathways mediate H3.3 de novo deposition and recycling during transcription. Nat. Struct. Mol. Biol. 27, 1057–1068. 10.1038/s41594-020-0492-7 32895554

[B156] Truch J. Telenius J. Higgs D. R. Gibbons R. J. (2018). How to tackle challenging ChIP-seq, with long-range cross-linking, using ATRX as an example. Methods Mol. Biol. 1832, 105–130. 10.1007/978-1-4939-8663-7_6 30073524

[B157] Tvardovskiy A. Schwämmle V. Kempf S. J. Rogowska-Wrzesinska A. Jensen O. N. (2017). Accumulation of histone variant H3.3 with age is associated with profound changes in the histone methylation landscape. Nucleic Acids Res. 45, 9272–9289. 10.1093/nar/gkx696 28934504 PMC5766163

[B158] van Kruijsbergen I. Hontelez S. Elurbe D. M. van Heeringen S. J. Huynen M. A. Veenstra G. J. C. (2017). Heterochromatic histone modifications at transposons in Xenopus tropicalis embryos. Dev. Biol. 426, 460–471. 10.1016/j.ydbio.2016.08.031 27639284 PMC5350053

[B159] Venkatesh S. Workman J. L. (2015). Histone exchange, chromatin structure and the regulation of transcription. Nat. Rev. Mol. Cell Biol. 16, 178–189. 10.1038/nrm3941 25650798

[B160] Viktorovskaya O. Chuang J. Jain D. Reim N. I. López-Rivera F. Murawska M. (2021). Essential histone chaperones collaborate to regulate transcription and chromatin integrity. Genes Dev. 35, 698–712. 10.1101/gad.348431.121 33888559 PMC8091981

[B161] Wang T. Liu Y. Edwards G. Krzizike D. Scherman H. Luger K. (2018). The histone chaperone FACT modulates nucleosome structure by tethering its components. Life Sci. Alliance 1, e201800107. 10.26508/lsa.201800107 30456370 PMC6238592

[B162] Wang Y.-R. Xu N.-X. Wang J. Wang X.-M. (2019). Kabuki syndrome: Review of the clinical features, diagnosis and epigenetic mechanisms. World J. Pediatr. 15, 528–535. 10.1007/s12519-019-00309-4 31587141

[B163] Wasylishen A. R. Sun C. Moyer S. M. Qi Y. Chau G. P. Aryal N. K. (2020). Daxx maintains endogenous retroviral silencing and restricts cellular plasticity *in vivo* . Sci. Adv. 6, eaba8415. 10.1126/sciadv.aba8415 32821827 PMC7406367

[B164] Wen H. Li Y. Xi Y. Jiang S. Stratton S. Peng D. (2014). ZMYND11 links histone H3.3K36me3 to transcription elongation and tumour suppression. Nature 508, 263–268. 10.1038/nature13045 24590075 PMC4142212

[B165] Wimmer M. E. Fant B. Swinford-Jackson S. E. Testino A. Nest D. V. Abel T. (2019). H3.3 barcoding of nucleus accumbens transcriptional activity identifies novel molecular cascades associated with cocaine self-administration in mice. J. Neurosci. 39, 5247–5254. 10.1523/JNEUROSCI.0015-19.2019 31043484 PMC6607753

[B166] Winkler D. D. Luger K. (2011). The histone chaperone FACT: Structural insights and mechanisms for nucleosome reorganization. J. Biol. Chem. 286, 18369–18374. 10.1074/jbc.R110.180778 21454601 PMC3099653

[B167] Wolf G. Rebollo R. Karimi M. M. Ewing A. D. Kamada R. Wu W. (2017). On the role of H3.3 in retroviral silencing. Nature 548, E1. 10.1038/nature23277 28770848 PMC6258051

[B168] Wong L. H. Ren H. Williams E. McGhie J. Ahn S. Sim M. (2009). Histone H3.3 incorporation provides a unique and functionally essential telomeric chromatin in embryonic stem cells. Genome Res. 19, 404–414. 10.1101/gr.084947.108 19196724 PMC2661805

[B169] Wong M. M. Cox L. K. Chrivia J. C. (2007). The chromatin remodeling protein, SRCAP, is critical for deposition of the histone variant H2A.Z at promoters. J. Biol. Chem. 282, 26132–26139. 10.1074/jbc.M703418200 17617668

[B170] Wright S. Finnegan D. (2001). Genome evolution: Sex and the transposable element. Curr. Biol. 11, R296–R299. 10.1016/s0960-9822(01)00168-3 11369217

[B171] Xu X. Ni K. He Y. Ren J. Sun C. Liu Y. (2021). LSH mediates gene repression through macroH2A deposition. Nat. Commun. 12, 5647. 10.1038/s41467-020-19159-0 33159050 PMC7648012

[B172] Xu Y. Ayrapetov M. K. Xu C. Gursoy-Yuzugullu O. Hu Y. Price B. D. (2012). Histone H2A.Z controls a critical chromatin remodeling step required for DNA double-strand break repair. Mol. Cell 48, 723–733. 10.1016/j.molcel.2012.09.026 23122415 PMC3525728

[B173] Yang B. X. El Farran C. A. Guo H. C. Yu T. Fang H. T. Wang H. F. (2015). Systematic identification of factors for provirus silencing in embryonic stem cells. Cell 163, 230–245. 10.1016/j.cell.2015.08.037 26365490 PMC4686136

[B174] Yilmaz D. Furst A. Meaburn K. Lezaja A. Wen Y. Altmeyer M. (2021). Activation of homologous recombination in G1 preserves centromeric integrity. Nature 600, 748–753. 10.1038/s41586-021-04200-z 34853474

[B175] Zeitlin S. G. Baker N. M. Chapados B. R. Soutoglou E. Wang J. Y. J. Berns M. W. (2009). Double-strand DNA breaks recruit the centromeric histone CENP-A. Proc. Natl. Acad. Sci. U. S. A. 106, 15762–15767. 10.1073/pnas.0908233106 19717431 PMC2747192

[B176] Zhang M. Zhao X. Feng X. Hu X. Zhao X. Lu W. (2022). Histone chaperone HIRA complex regulates retrotransposons in embryonic stem cells. Stem Cell Res. Ther. 13, 137. 10.1186/s13287-022-02814-2 35365225 PMC8973876

[B177] Zhang R. Poustovoitov M. V. Ye X. Santos H. A. Chen W. Daganzo S. M. (2005). formation of MacroH2A-containing senescence-associated heterochromatin foci and senescence driven by ASF1a and HIRA. Dev. Cell 8, 19–30. 10.1016/j.devcel.2004.10.019 15621527

[B178] Zhou K. Liu Y. Luger K. (2020). Histone chaperone FACT FAcilitates chromatin transcription: Mechanistic and structural insights. Curr. Opin. Struct. Biol. 65, 26–32. 10.1016/j.sbi.2020.05.019 32574979

[B179] Zovkic I. B. Paulukaitis B. S. Day J. J. Etikala D. M. Sweatt J. D. (2014). Histone H2A.Z subunit exchange controls consolidation of recent and remote memory. Nature 515, 582–586. 10.1038/nature13707 25219850 PMC4768489

