# Supplementary material for: Histones and their chaperones: Adaptive remodelers of an ever-changing chromatinic landscape
Source: Front Genet. 2022 Nov 16;13:1057846. doi: 10.3389/fgene.2022.1057846 (PMC9709290; doi:10.3389/fgene.2022.1057846)
Supplement: Supplementary file 1 [file Table1.DOCX]

# Supplementary Table S1. Reported regulators of transposon activity: histone variants, histone marks, histone chaperones and other interactors

| **Class 1 transposons (retrotransposons)** | | | | **Incorporated histones** | **Associated histone marks** | **Associated chaperones** | **Other associated interactors** | **Cell type** | **References** |
| --- | --- | --- | --- | --- | --- | --- | --- | --- | --- |
| **Subclass** | **Superfamily** | **Family** | **Subfamily** |  |  |  |  |  |  |
| LTR | ERV | ERV I | MuLV/RLTR4 | H3.3–H4 | H3K9me2  H3K9me3 | HIRA | ASF1A | Mouse E14 ESCs | Zhang et al. (2022) |
|  |  |  |  | − | H3K9me3 | − | ESET  TRIM28 | Mouse ESCs | Matsui et al. (2010) |
|  |  | ERV II | ETn/MusD | H3.3–H4 | H3K9me3  H3K27me3 | ATRX–DAXX | ESET | Mouse ESCs and E13.5 PGCs | Liu et al. (2014)  Elsässer et al. (2015) |
|  |  |  |  | − | H3K9me3 | − | ESET  TRIM28 | Mouse ESCs | Matsui et al. (2010) |
|  |  |  | ERVK10C | H3.3 | H3K9me3  H3K27me3 | − | ESET | Mouse E13.5 PGCs | Liu et al. (2014) |
|  |  |  | MMERVK | H3.3–H4 | H3K9me3 | ATRX–DAXX | SUV39H1 | Mouse ESCs | He et al. (2015) |
|  |  |  | IAP | H3.3–H4 | H3K9me3 | ATRX–DAXX | ESET | Mouse ESCs | Elsässer et al. (2015) |
|  |  |  |  | H3.3–H4 | H3K9me3 | ATRX–DAXX | ESET  TRIM28 | Mouse ESCs | Sadic et al. (2015) |
|  |  |  |  | H3.1/H3.2–H4 | H3K9me3 | CAF-1 | KMT5B (SUV420H1)  KMT5C (SUV420H2) | Preimplantation mouse embryos | Hatanaka et al. (2015) |
|  |  |  |  | − | H3K9me3 | − | ESET  TRIM28 | Mouse ESCs | Matsui et al. (2010) |
|  |  |  |  | − | H3K9me3 | − | TRIM28 | Mouse ESCs | Rowe et al. (2010, 2013) |
|  |  |  |  | H3.3–H4 | H3K9me3 | ATRX–DAXX | SUV39H1 | Mouse ESCs | He et al. (2015) |
|  |  |  |  | H3.3–H4 | H3K9me2  H3K9me3 | HIRA | ASF1A | Mouse E14 ESCs | Zhang et al. (2022) |
|  |  | ERV III | MERVL | H2A/H2B | H2Bub | FACT | USP7 | Mouse E14 and J1 ESCs | Chen et al. (2020) |
|  |  |  |  | H3.1/H3.2–H4 | H3K4me2 H3K9Ac  H3K27Ac | CAF-1 | CHAF1A  TRIM28  ESET  SUMO2 | Mouse ESCs | Yang et al. (2015) |
|  |  |  |  | H3.3–H4 | H3K9me2  H3K9me3 | HIRA | ASF1A | Mouse E14 ESCs | Zhang et al. (2022) |
| Non-LTR | LINE | LINE-1 | | H3.1/H3.2–H4 | H3K9me2  H3K9me3  H3K27me3  H4K20me3 | CAF-1 | KMT5B (SUV420H1)  KMT5C (SUV420H2) | Preimplantation mouse embryos | Hatanaka et al. (2015) |
|  | SINE | SINE-B2 | | H3.1/H3.2–H4 | H3K9me2  H3K9me3  H3K27me3  H4K20me3 | CAF-1 | KMT5B (SUV420H1)  KMT5C (SUV420H2) | Preimplantation mouse embryos | Hatanaka et al. (2015) |

For more information on family-level classification of transposons, see the Dfam database resource (Storer et al., 2021).

**Acronyms and symbols**: LTR, long terminal repeat retrotransposon; Non-LTR, Non-long terminal repeat retrotransposon; ERV, endogenous retrovirus-like sequence; LINE, long interspersed nuclear element; SINE, short interspersed nuclear element; MuLV/RLTR4, murine leukemia virus (internal sequence/LTR); ETn/MusD, early transposon/Mus type-D related retrovirus; ERVK10C, endogenous retrovirus with lysine tRNA primer (ERV II) corresponding to RLTR10C; MMERVK, mouse endogenous retrovirus with lysine tRNA primer (ERV II); IAP, intracisternal A-particle retrotransposon; MERVL, murine endogenous retrovirus with leucine tRNA primer (ERV III); LINE-1, L1 LINE family; SINEB2, B2 SINE family; HIRA, histone cell cycle regulator (protein or complex); ESET, histone-lysine N-methyltransferase SETDB1; TRIM28, transcription intermediary factor 1-beta (also KAP-1); ATRX, transcriptional regulator ATRX; DAXX, death domain-associated protein 6; FACT, facilitates chromatin transcription (complex); CAF-1, chromatin assembly factor 1 (complex including CHAF1A subunit); ASF1A, histone chaperone ASF1A (anti-silencing function protein 1 homolog A); USP7, ubiquitin carboxyl-terminal hydrolase 7 (UBP7); SUMO2, small ubiquitin-related modifier 2; SUV39H1, histone-lysine N-methyltransferase SUV39H1 (suppressor of variegation 3-9 homolog 1); KMT5B, histone-lysine N-methyltransferase KMT5B (also SUV420H1: suppressor of variegation 4-20 homolog 1); KMT5C, histone-lysine N-methyltransferase KMT5C (also SUV420H2: suppressor of variegation 4-20 homolog 2); ESCs, embryonic stem cells; PGCs, primordial germ cells. The nomenclature of histone modifications/marks: in the case of H3K9me3, H3 refers to the core histone protein, K refers to the amino acid, the number 9 indicates the position of lysine residue and me3 refers to the type of modification (Prakash and Fournier, 2018).

# Supplementary References

Chen, F., Zhang, W., Xie, D., Gao, T., Dong, Z., and Lu, X. (2020). Histone chaperone FACT represses retrotransposon MERVL and MERVL-derived cryptic promoters. *Nucleic Acids Res.* 48, 10211–10225. doi: 10.1093/nar/gkaa732.

Elsässer, S. J., Noh, K.-M., Diaz, N., Allis, C. D., and Banaszynski, L. A. (2015). Histone H3.3 is required for endogenous retroviral element silencing in embryonic stem cells. *Nature* 522, 240–244. doi: 10.1038/nature14345.

Hatanaka, Y., Inoue, K., Oikawa, M., Kamimura, S., Ogonuki, N., Kodama, E. N., et al. (2015). Histone chaperone CAF-1 mediates repressive histone modifications to protect preimplantation mouse embryos from endogenous retrotransposons. *Proc. Natl. Acad. Sci.* 112, 14641–14646. doi: 10.1073/pnas.1512775112.

He, Q., Kim, H., Huang, R., Lu, W., Tang, M., Shi, F., et al. (2015). The Daxx/Atrx Complex Protects Tandem Repetitive Elements during DNA Hypomethylation by Promoting H3K9 Trimethylation. *Cell Stem Cell* 17, 273–286. doi: 10.1016/j.stem.2015.07.022.

Liu, S., Brind’Amour, J., Karimi, M. M., Shirane, K., Bogutz, A., Lefebvre, L., et al. (2014). Setdb1 is required for germline development and silencing of H3K9me3-marked endogenous retroviruses in primordial germ cells. *Genes Dev.* 28, 2041–2055. doi: 10.1101/gad.244848.114.

Matsui, T., Leung, D., Miyashita, H., Maksakova, I. A., Miyachi, H., Kimura, H., et al. (2010). Proviral silencing in embryonic stem cells requires the histone methyltransferase ESET. *Nature* 464, 927–931. doi: 10.1038/nature08858.

Prakash, K., and Fournier, D. (2018). Evidence for the implication of the histone code in building the genome structure. *Biosystems* 164, 49–59. doi: 10.1016/j.biosystems.2017.11.005.

Rowe, H. M., Jakobsson, J., Mesnard, D., Rougemont, J., Reynard, S., Aktas, T., et al. (2010). KAP1 controls endogenous retroviruses in embryonic stem cells. *Nature* 463, 237–240. doi: 10.1038/nature08674.

Rowe, H. M., Kapopoulou, A., Corsinotti, A., Fasching, L., Macfarlan, T. S., Tarabay, Y., et al. (2013). TRIM28 repression of retrotransposon-based enhancers is necessary to preserve transcriptional dynamics in embryonic stem cells. *Genome Res.* 23, 452–461. doi: 10.1101/gr.147678.112.

Sadic, D., Schmidt, K., Groh, S., Kondofersky, I., Ellwart, J., Fuchs, C., et al. (2015). Atrx promotes heterochromatin formation at retrotransposons. *EMBO Rep.* 16, 836–850. doi: 10.15252/embr.201439937.

Storer, J., Hubley, R., Rosen, J., Wheeler, T. J., and Smit, A. F. (2021). The Dfam community resource of transposable element families, sequence models, and genome annotations. *Mob. DNA* 12, 2. doi: 10.1186/s13100-020-00230-y.

Yang, B. X., EL Farran, C. A., Guo, H. C., Yu, T., Fang, H. T., Wang, H. F., et al. (2015). Systematic Identification of Factors for Provirus Silencing in Embryonic Stem Cells. *Cell* 163, 230–245. doi: 10.1016/j.cell.2015.08.037.

Zhang, M., Zhao, X., Feng, X., Hu, X., Zhao, X., Lu, W., et al. (2022). Histone chaperone HIRA complex regulates retrotransposons in embryonic stem cells. *Stem Cell Res. Ther.* 13, 137. doi: 10.1186/s13287-022-02814-2.
